# Deconvolution of ferredoxin, plastocyanin, and P700 transmittance changes in intact leaves with a new type of kinetic LED array spectrophotometer

**DOI:** 10.1007/s11120-016-0219-0

**Published:** 2016-02-02

**Authors:** Christof Klughammer, Ulrich Schreiber

**Affiliations:** Julius-von-Sachs Institut für Biowissenschaften, Universität Würzburg, Julius-von-Sachs Platz 2, 97082 Würzburg, Germany

**Keywords:** Chlorophyll fluorescence, Cyclic electron transport, FeS proteins, Flash relaxation kinetics, Photosystem I, Polyphasic fluorescence rise, Thioredoxin

## Abstract

A newly developed compact measuring system for assessment of transmittance changes in the near-infrared spectral region is described; it allows deconvolution of redox changes due to ferredoxin (Fd), P700, and plastocyanin (PC) in intact leaves. In addition, it can also simultaneously measure chlorophyll fluorescence. The major opto-electronic components as well as the principles of data acquisition and signal deconvolution are outlined. Four original pulse-modulated dual-wavelength difference signals are measured (785–840 nm, 810–870 nm, 870–970 nm, and 795–970 nm). Deconvolution is based on specific spectral information presented graphically in the form of ‘Differential Model Plots’ (DMP) of Fd, P700, and PC that are derived empirically from *selective* changes of these three components under appropriately chosen physiological conditions. Whereas information on *maximal* changes of Fd is obtained upon illumination after dark-acclimation, *maximal* changes of P700 and PC can be readily induced by saturating light pulses in the presence of far-red light. Using the information of DMP and maximal changes, the new measuring system enables *on*-*line* deconvolution of Fd, P700, and PC. The performance of the new device is demonstrated by some examples of practical applications, including fast measurements of flash relaxation kinetics and of the Fd, P700, and PC changes paralleling the polyphasic fluorescence rise upon application of a 300-ms pulse of saturating light.

**Electronic supplementary material:**

The online version of this article (doi:10.1007/s11120-016-0219-0) contains supplementary material, which is available to authorized users.

## Introduction

All reducing power of phototrophic life on earth originates from the light-driven oxidation of special chlorophyll molecules in the reaction centers of the two photosystems, PS I and PS II. While PS II generates a strong oxidant, providing the oxidizing power for the splitting of water, PS I generates a strong reductant capable of reducing NADP, with reduced iron–sulfur proteins being the first stable PS I electron acceptors (Malkin and Bearden 1971; Hiyama and Ke 1971). PS II is normally limited by the availability of oxidized electron acceptors, since the re-reduction of the primary donor, P680, is faster than the reoxidation of the first stable acceptor *Q*_A_. Hence, the redox state of *Q*_A_ may serve as an indicator of the ‘openness’ of PS II, which can be readily assessed via measurements of variable Chl fluorescence (Kautsky et al. 1960; Duysens and Sweers 1963; Melis and Duysens 1979; Joliot and Joliot 1979; Melis and Schreiber 1979; Papageorgiou and Govindjee 2004). In contrast, depending on conditions, PS I can be limited by the donor as well as by the acceptor side (Klughammer and Schreiber 1994), but it does not show variable Chl fluorescence under physiological conditions. When the primary (electron) donor, P700, is reduced, excitation energy is quenched photochemically, whereas it is quenched non-photochemically when it is oxidized. Hiyama and Ke (1971) discovered the spectral species ‘P430’, with characteristics of a primary electron acceptor of PS I, which later was identified as terminal [4Fe–4S] cluster of PS I (Oh-Oka et al. 1991; Hoshina and Itoh 1993). Spectral changes of P430 can be distinguished from those of the much larger P700 changes in vitro only when donor and acceptor sides of PS I can be controlled by chemical additions. Therefore, P430 absorbance changes have not been used for assessment of PS I activity in vivo.

PS I is an integral membrane protein complex that transfers electrons from the soluble electron carrier plastocyanin, PC, at the lumenal side of the thylakoid membrane to the soluble electron carrier ferredoxin, Fd, at the stromal side (for reviews see Golbeck 2006; Jensen et al. 2007). Within the PS I core complex charge separation is stabilized via a cascade of rapid electrons transfer reactions in the ps to ns time range from P700 via A_0_ (monomeric form of Chl *a*) and A_1_ (phylloquinone) to the [4Fe–4S] centers *F*_X_, *F*_A_, and *F*_B_, with the latter corresponding to P430 of Hiyama and Ke (1971). *F*_B_ is the distal stromal FeS cluster that transfers electrons to the soluble [2Fe–2S] cluster Fd (Vassiliev et al. 1998; Diaz-Quintana et al. 1998). In the absence of oxidized Fd or any other exogenous electron acceptor, the formation of the charge-separated state P700^+^*F*_B_^−^ is followed by charge recombination occurring in the 10–100 ms time range (Brettel 1997). Fd is a small cluster protein (ca. 11 kDa) ligated by four cysteine residues that can bind with high affinity to the stromal side of PS I (Sétif 2001), serving as a one-electron carrier (E_m_ ~ −420 mV). Sétif and Bottin (1995) reported on first-order half-times of Fd reduction in the sub-µs and µs ranges and half-times corresponding to second-order processes in the range of a few hundred µs, based on laser flash-absorption spectroscopy in the visible region using PS I particles and externally added Fd. These in vitro measurements took advantage of the fact that in the 460–600 nm spectral region reduction induced absorption changes of the [2Fe–2S] cluster of Fd differ from those of the terminal intra-membrane [4Fe–4S] clusters *F*_A_ and *F*_B_ (Sétif 2001). Analogous in vivo measurements differentiating between Fd and *F*_A_ − *F*_B_ reduction may be considered practically impossible, because of the much larger absorption changes caused by numerous other components. However, in many applications, including most measurements presented in the present report, such differentiation is not essential, so that the *entity of all FeS proteins* may be considered as ‘PS I acceptor’ which for the sake of simplicity will be referred to as ‘Fd’ in the present report. In view of the substantial differences in redox potentials, we may expect that under in vivo conditions of PS I acceptor side limitation, electrons will first accumulate in the pool of soluble Fd before the lifetime of reduced [4Fe–4S] clusters is increased.

Besides binding close to *F*_B_ to the PS I complex, soluble Fd can also bind to various stromal enzymes and thus plays a unique role as *distributor of reducing power* between numerous metabolic pathways (for a review see Hanke and Mulo 2013). These include the ferredoxin-NADP reductase (FNR) catalyzed reduction of NADP (Shin et al. 1963; Carillo and Ceccarelli 2003), reduction of O_2_ and H_2_O_2_ in the Mehler-ascorbate peroxidase (MAP) cycle (Asada and Badger 1984; Schreiber et al. 1995; Asada 1999; Miyake 2010), nitrite reduction (Anderson and Done 1978), various types of cyclic electron transport (CET) (Arnon and Chain 1975; Bendall and Manasse 1995; Miyake et al. 1995; Munekage et al. 2002), and reduction of thioredoxin, the key redox regulator of numerous processes at various levels of chloroplast metabolism (Buchanan 1980; Knaff 1996; Buchanan et al. 2002; Schürmann and Buchanan 2008).

In spite of its pivotal role in in vivo photosynthesis, so far no instrumentation has been available to measure Fd redox changes in intact organisms under in vivo conditions. Redox changes of Fd are accompanied by relatively small absorbance changes in the visible and near-infrared (NIR) which, however, are overlapped by much larger changes of P700 and other components and, hence, have proven difficult to be reliably measured, particularly under in vivo conditions. Klughammer and Schreiber (1991) first analyzed NIR absorption changes of Fd in *intact* spinach chloroplasts. Fd reduction causes an increase of NIR-transmittance that declines from 780 to 900 nm (Klughammer 1992; see also Supplementary Figs. 1, 3, 4). It thus simulates P700 reduction which causes a broad peak of bleaching around 790–830 nm declining towards 900 nm (Schreiber et al. 1988; see also Supplementary Figs. 2, 4). Klughammer (1992) extended the Fd transmittance measurements on intact spinach leaves using the 771–834 nm difference signal, which is relatively selective for Fd, as the combined differential changes of P700 and plastocyanin (PC) are equal at these two wavelengths and an electrochromic pigment shift peaking around 730 nm (Klughammer and Schreiber 1991) is negligibly small above 765 nm.

NIR-absorbance changes of P700 measured by the pulse-amplitude modulation technique have become a widespread non-intrusive tool for assessment of PS I quantum yield and electron transport reactions at PS I donor and acceptor sides (Weis et al. 1987; Harbinson and Woodward 1987; Schreiber et al. 1988; Harbinson and Hedley 1993; Siebke et al. 1991; Harbinson and Foyer 1991; Asada et al. 1992; Foyer et al. 1992; Laisk et al. 1992; Klughammer and Schreiber 1994, 1998; Sacksteder and Kramer 2000; Bukhov et al. 2002; Joliot and Joliot 2002; Oja et al. 2003; Schansker et al. 2003; Golding and Johnson 2003; Oja et al. 2004; Chow and Hope 2004; Talts et al. 2007; Laisk et al. 2010). The above-cited work is just a small selection of many more publications on ‘P700’ changes, most of which were carried out with commercially available devices that do *not* allow to differentiate between P700, PC, and Fd changes. While this work has generated an enormous amount of information on photosynthetic electron transport, part of this information should be regarded with some caution: When possible contributions of PC and Fd are not taken into account, this in some cases may lead to erroneous conclusions. In a spinach leaf, the maximal P700 absorbance change in the 790–840 nm range is about 2× larger than that of PC and about 4× larger than that of Fd (Klughammer 1992). Hence, while P700 changes are dominant, the potential contributions of PC and Fd are substantial. Whether these contributions may be considered negligibly small or not, depends on the kind of sample, the physiological conditions, the employed measuring technique and the particular question to be answered by the experiment. Whereas in some applications it is not essential whether oxidation of PS I donors refers to (P700 + PC) or specifically to P700, this distinction can be of utmost importance e.g., for the determination of PS I quantum yield (Oja et al. 2003).

Besides its major absorption peak at 597 nm (Katoh 1977) PC displays a minor absorption peak in the NIR around 770 nm. The decline of PC absorption above 830 nm is distinctly less steep than in the case of P700 (Klughammer and Schreiber 1991; see also Supplementary Figs. 1, 2, 4). This difference can be used for deconvolution of PC and P700 changes. Kirchhoff et al. (2004) applied two single-beam PAM spectrophotometers (pulse-modulated measuring light peaking at 810 and 870 nm) for the deconvolution of P700 and PC transmittance changes in isolated spinach thylakoid membranes. The differential absorption coefficients required for deconvolution were determined from PS I- or PC-enriched preparations and the experiments were carried out in the presence of the artificial PS I acceptor methyl viologen and of uncouplers, thus avoiding the interference of changes other than P700 and PC. In this way, Kirchhoff et al. (2004) were able to study the redox equilibration between PC and P700 under a variety of conditions, showing that equilibration between the components of the ‘high potential chain’ is not homogeneous throughout the membrane. Using essentially the same rationale as Kirchhoff et al. (2004), Schöttler et al. (2007a, b) and Aronsson et al. (2008) employed a special PAM spectrophotometer measuring 830–870 nm and 870–950 nm difference signals for the deconvolution of P700 and PC redox changes and for the determination of the relative stoichiometries of PC per P700.

Deconvolution of P700 and PC changes from NIR-transmittance signals was also carried out by Oja et al. (2003) and Laisk et al. (2010) based on experimentally determined redox equilibrium constants for P700/PC and algorithms that assume homogeneous redox equilibration within the ‘high potential chain’. Laisk et al. (2010) simultaneously measured single-wavelength transmittance at 810 and 950 nm in sunflower leaves and determined the ratios of the extinction coefficients of P700 and PC at these two wavelengths from the post-FR-illumination transients of the two signals, putting forward arguments that under the chosen conditions full equilibration can be assumed.

In all this previous work on P700 and PC, with or without deconvolution of these two components, Fd transmittance changes were considered negligibly small, so that it was assumed that their contribution can be ignored. So far, except for the early studies of Klughammer and Schreiber (1991) and Klughammer (1992) no attempts have been made to ‘harvest’ information contained in these changes for analyzing the state of the PS I acceptor side and the Fd-driven reactions, mainly due to the expected difficulties of separating the relatively small changes of Fd from the distinctly larger changes of P700 and PC. Here we report on a newly developed measuring system with exceptional sensitivity and selectivity, with which these difficulties were overcome. This system employs four dual-wavelength difference signals for the deconvolution of Fd, P700, and PC. This was derived from a previously developed ‘Kinetic LED-Array Spectrophotometer’ (KLAS-100) for time-resolved measurements in the 505–570 nm spectral range and deconvolution of cyt f, cyt b6, cyt b559, and C550 redox changes from much larger changes of P515 (ECS), ‘scattering’ and zeaxanthin (Klughammer et al. 1990). A peculiarity of the new measuring system, which was adopted from the KLAS-100, is the *empirical approach of deconvolution* based on the spectral information obtained under appropriately chosen conditions upon selective changes of single components. The measuring technique and methodology of deconvolution will be outlined below and some examples of application are presented.

## Materials and methods

### Device for simultaneous measurements of NIR-absorbance and Chl *a* fluorescence changes

**Fig. 1:**
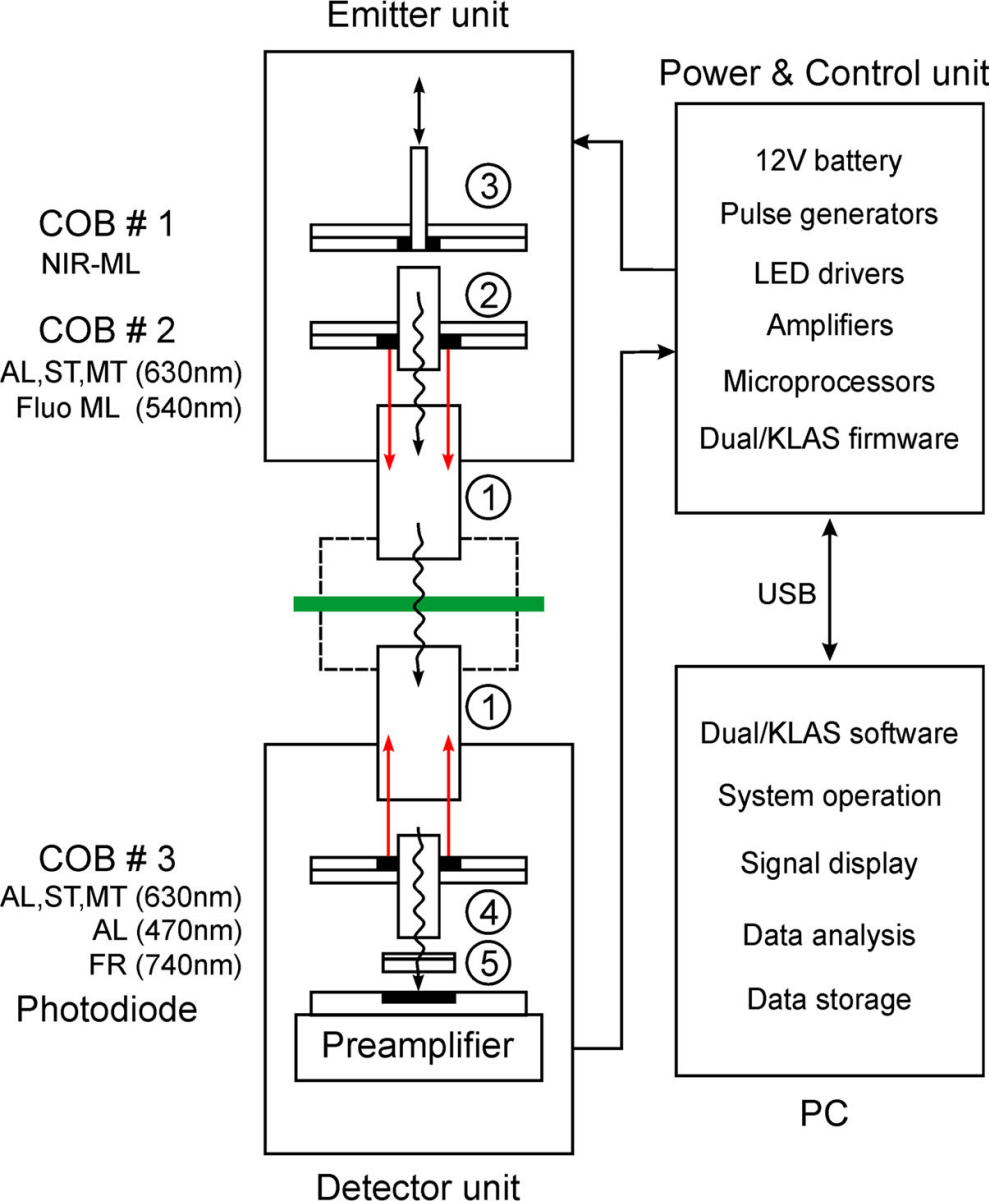
Schematic view of the major components of the newly developed measuring system. At the core of the Emitter unit is the chip-on-board LED array COB #1 which provides the NIR-ML for the four difference signals. Actinic illumination (AL) single and multiple turnover flashes (ST and MT) are applied at both sides of the *green leaf* using COBs #2 and #3 in the Emitter and Detector units, respectively. The various light qualities are guided and randomly mixed via *Perspex* rods (*1*–*4*). The transmitted light passes the filter set (*5*) eliminating wavelengths <720 nm. For further explanations see text

Figure 1 shows a block diagram describing the essential parts of the new device for simultaneous measurements of NIR-transmittance and Chl *a* fluorescence changes. The optical part consists of Emitter and Detector units, both of which are equipped with 10 × 10 mm *Perspex* light guides **(1)** between which the leaf sample is sandwiched. The optics is compatible with the optional 3010-DUAL cuvette for leaf measurements with the DUAL-PAM-100 and GFS-3000 gas exchange measuring system (Heinz Walz GmbH, Effeltrich, Germany). For the basic measurements described in this communication a standard leaf clip was used, where the leaf is held between two sponge rubber rings, with 1 mm air space on both sides between leaf and the two *Perspex* light-guide rods. Within the Emitter unit the other side of the light guide is illuminated by two separate chip-on-board (COB) LED arrays with near-infrared measuring light (COB #1, NIR-ML) and 630 nm actinic light (COB #2, AL, ST, and MT). The NIR-ML is guided via a 6.5-mm *Perspex* rod **(2)** through a 6.5-mm central hole in COB #2 towards the 10 × 10 *Perspex* light guide **(1).** In addition, a single 540 nm LED is provided for pulse-modulated fluorescence measuring light (Fluo ML). The same LED array serving for continuous actinic illumination (AL) can also be employed for single and multiple turnover flashes (ST and MT, respectively).

**Fig. 2:**
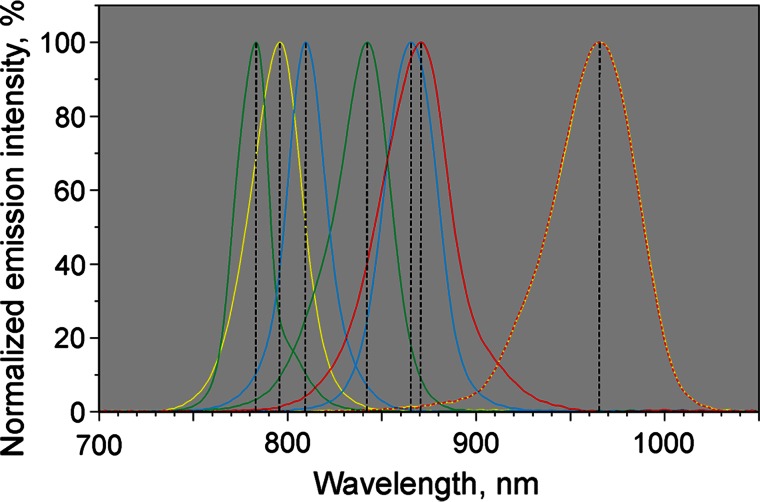
Normalized emission spectra of the four NIR-ML wavelength pairs employed for measuring the four transmittance difference signals 785–840 nm (*green* ‘Fd’), 795–970 nm (*yellow* ‘All’), 810–870 nm (*blue* ‘P700’), and 870–970 nm (*red* ‘PC’). See text for further explanations

The NIR-ML COB LED array features Power-LED Chips for generation of eight kinds of single-wavelength pulse-modulated NIR-ML peaking at 785, 795, 810, 840 nm, 2× 870 nm, and 2× 970 nm, which form the wavelength pairs 785–840 nm (‘Fd’), 795–970 nm (‘All’), 810–870 nm (‘P700’), and 870–970 nm (‘PC’) (see below). The 785 nm ML is filtered via a miniature (2 × 2 × 0.6 mm) 20 nm band-pass interference filter, effectively eliminating wavelengths below 775 nm, which is mounted directly on top of the LED chip. Likewise also the ‘P700’ chips (810 and 870 nm) are equipped with miniature 20 nm band-pass filters. The spectra of the four pairs of NIR-ML are shown in Fig. 2. Due to the partial use of filters the spectra differ in shape and the peak wavelengths do not necessarily correspond to the center of emission.

COB #1 contains a 2.5-mm central hole, through which optionally, via a 2.5-mm *Perspex* rod **(3)** additional light (e.g., multi-color fluorescence measuring light or a laser flash) may be applied or fluorescence from the leaf surface may be detected. For *standard* fluorescence measurements the same photodiode detector as for transmitted NIR-ML in the Detector unit is employed.

In the Detector unit the light guide faces another COB LED Array equipped with 630 nm Power-LED Chips for red AL, ST, and MT (COB #3), identical to the array employed in the Emitter unit. In addition also 470 nm LEDs for blue AL (e.g., for inducing stomatal opening) and 740 nm LEDs for far-red illumination (FR) are provided. The COB metal core board features a central 6.5-mm hole through which the transmitted NIR-ML as well as Chl *a* fluorescence are guided via a 6.5-mm *Perspex* rod **(4)** and the detector filter set **(5)** to a 10 × 10 mm PIN photodiode detector. The filter set consists of a 715-nm long-pass glass filter (3 mm RG9, Schott) protecting the photodiode against visible light, in front of which a non-fluorescing plastic filter (Lee #27, Medium Red) is mounted that absorbs transmitted 540 nm Fluo ML which otherwise would create some fluorescence background signal by excitation of RG9 filter fluorescence. The photodiode serves for detection of the eight pulse-modulated NIR-transmittance signals and of pulse-modulated Chl *a* fluorescence in a time-sharing mode with *standard* 1 ms time resolution when all signals are measured simultaneously. The photodiode is directly mounted on the preamplifier board.

After preamplification, four pulse-modulated transmittance difference signals (785–840 nm, 810–870 nm, 870–970 nm, and 795–970 nm) as well as the pulse-modulated fluorescence signal are processed with the help of selective window amplifiers within the DUAL/KLAS-NIR Power & Control unit. The latter is equipped with a 12-V battery. It contains the LED driver boards with the pulse generators, the microprocessors, with which pulse generation and signal detection are controlled, as well as the selective window amplifiers for selective amplification of the four pulse-modulated NIR-transmittance difference signals and up to two pulse-modulated fluorescence signals. Part of signal processing is controlled by dedicated Dual/KLAS firmware.

An external computer with high processing power (in conjunction with the dedicated Dual/KLAS software) is an essential part of the new measuring system. It serves for system operation, signal display, data analysis, and data storage. Communication between PC and the Power & Control unit is via USB interface. Various modes of operation are provided for the recording of fast and slow signal changes, including the possibility to measure each of the four dual-wavelength difference signals separately at correspondingly higher time resolution.

Two settings of hardware signal damping are provided for fast and slow kinetics measurements, with 30 µs and 1 ms time constants, respectively. Saved data can be further processed by point averaging (software damping). The software supports repetitive measurements with on-line and off-line averaging.

### Principles of data acquisition and analysis

**Fig. 3:**
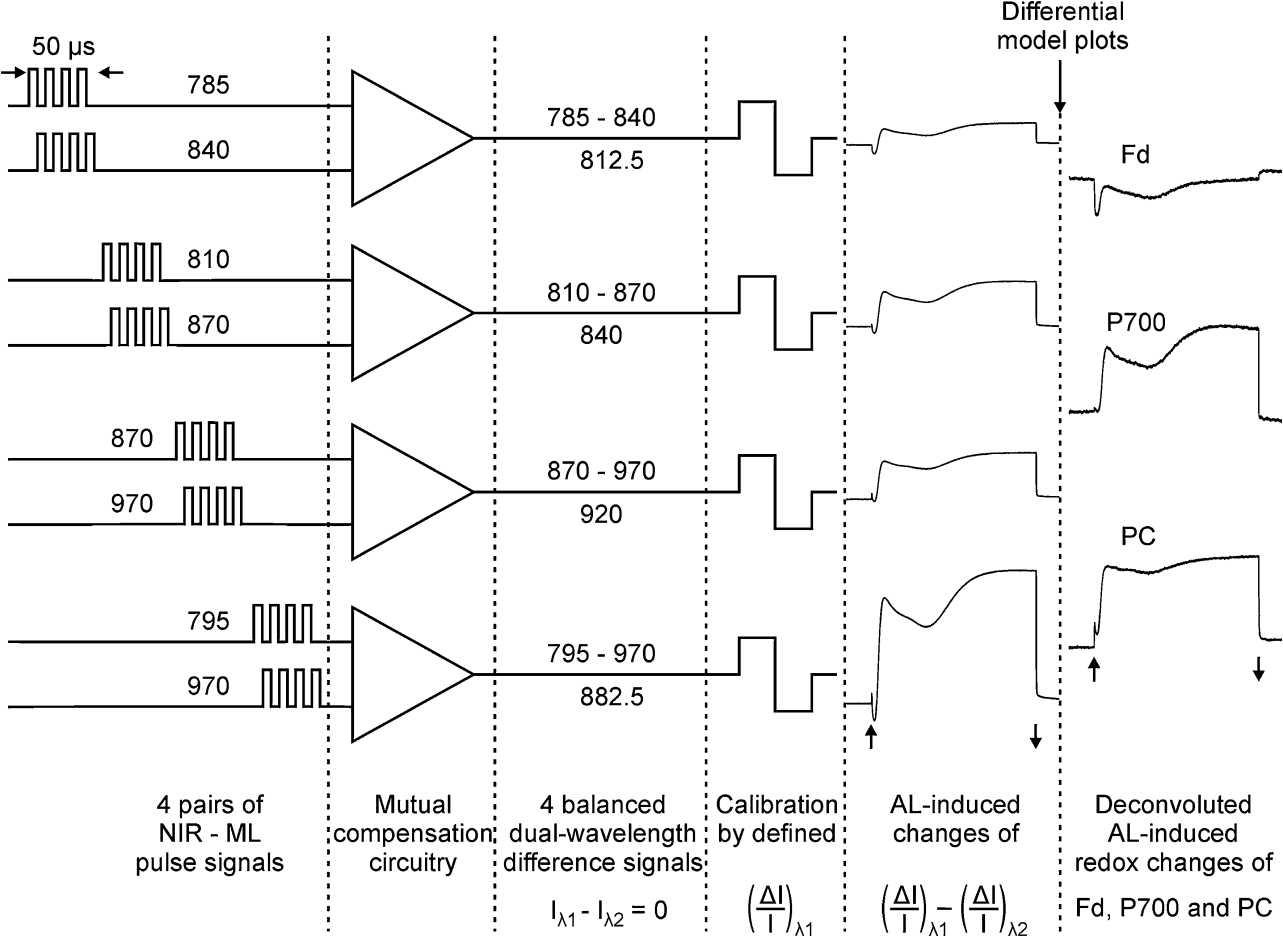
Scheme outlining data acquisition and deconvolution. Transmittance signals originating from four pairs of pulse-modulated NIR-ML are zeroed and calibrated before recording of light-induced changes. On-line deconvolution of Fd, P700, and PC redox changes is based on ‘differential model plots’ saved in computer memory. See text for further explanations

**Fig. 4:**
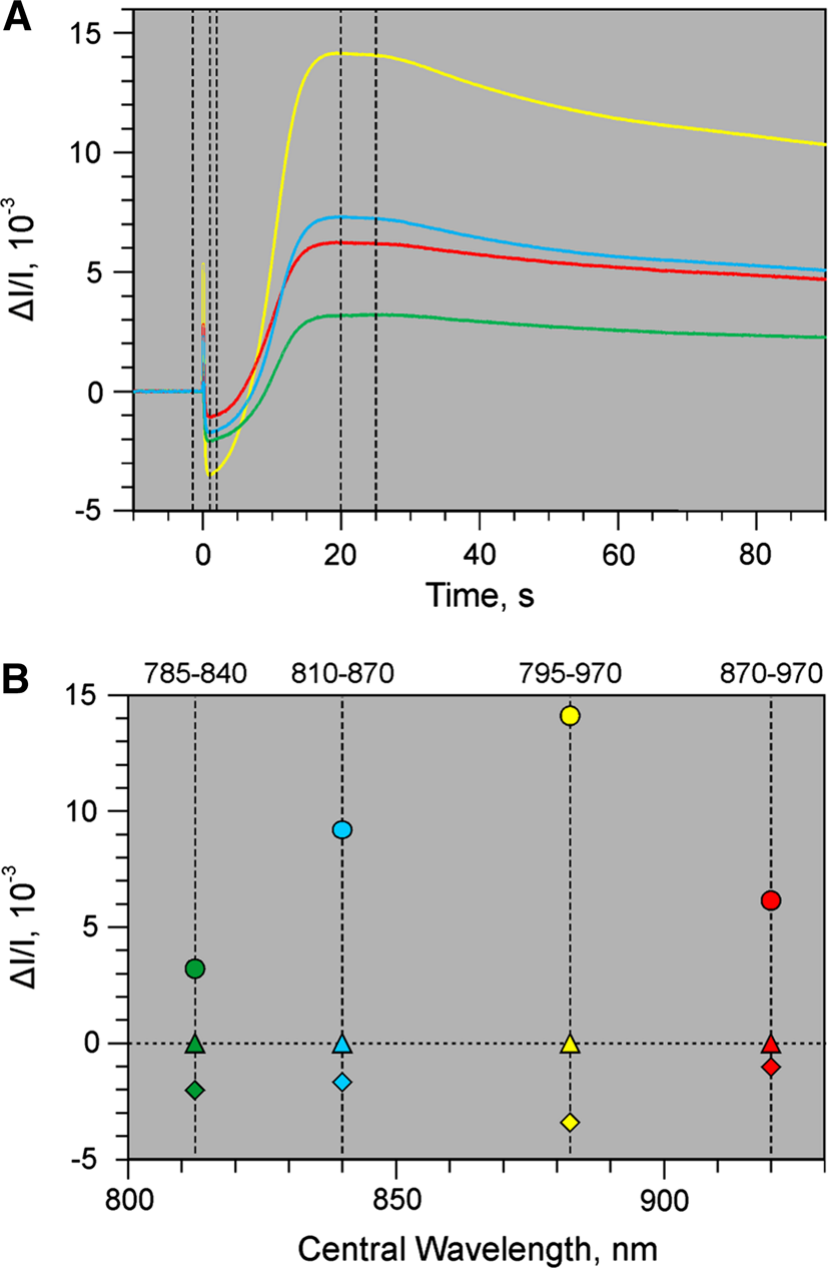
Light-induced changes of the four transmittance difference signals 785–840 nm (*green*), 795–970 nm (*yellow*), 810–870 nm (*blue*), and 870–970 nm (*red*). Dark-adapted ivy leaf, 300 µmol photons m^−2^s^−1^ (630 nm) actinic illumination. **a** Original kinetic recordings (single measurement). *Broken vertical lines* refer to time windows defining the zero baseline (before AL-on) and the sampling periods (1–2 s and 20–25 s), over which the Δ*I*/*I* values are averaged that are used for the ‘differential plots’ in panel **b**. **b** Graphical presentation of the two sets of averaged values derived for the two time periods defined in panel **a** in the form of ‘differential plots’: positive values for 20–25 s window, negative values for 1–2 s window. The averaged Δ*I*/*I* values of the four difference signals are plotted against the central wavelengths of the four wavelength pairs

**Fig. 5:**
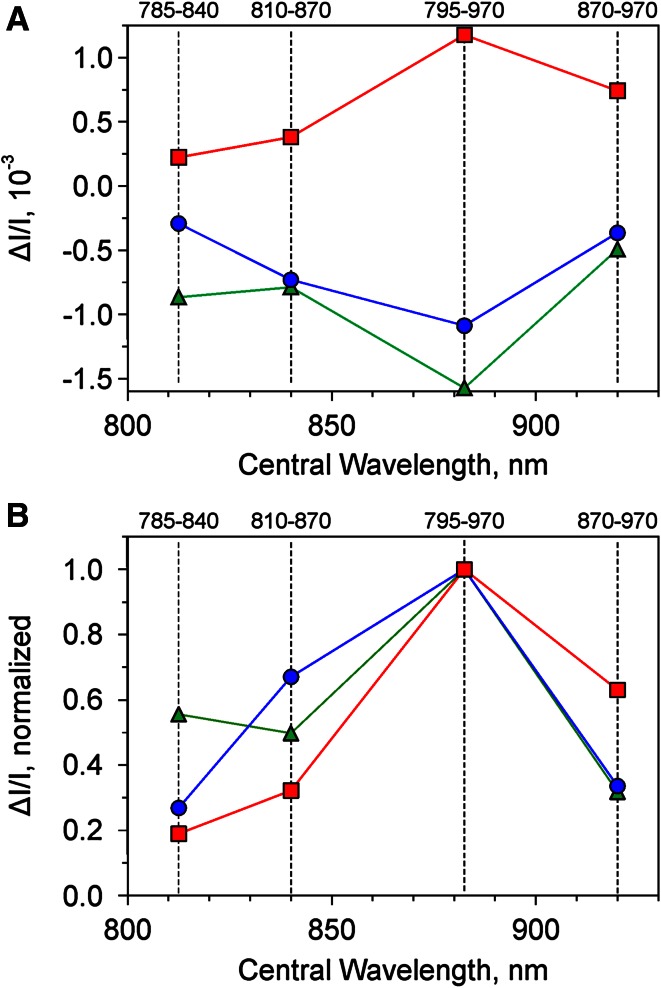
*Selective* dual-wavelength difference transmittance changes in a *dark-green leaf* of *Hedera helix* of Fd (*green*), P700 (*blue*), and PC (*red*) measured with the new device under the specific conditions outlined in the text, presented in the form of ‘differential plots’. Central wavelength refers to the mean values of the wavelengths of the four pairs indicated by the *four broken vertical lines*. **a** Original values of Δ*I*/*I* describing selective reduction of Fd, selective reduction of P700, and selective oxidation of PC. The original measurements of these selective changes will be presented below under ‘Results and discussion’ (Figs. 6, 7, 8). **b** Normalized values of Δ*I*/*I* derived from the original values in panel A, representative for selective *oxidation* of Fd, P700, and PC. The spectral information contained in this normalized plot (called *differential model plot*) is applied for *deconvolution* of redox changes of Fd, P700, and PC with the new device

The principles of data acquisition and analysis leading to deconvolution of Fd, P700, and PC absorbance changes with the new device are shown schematically in Fig. 3. Data acquisition is based on a rapid sequence of 2.5 µs pulses of NIR-ML, the timing of which is programed such that pairs of pulses of 785 and 840 nm, 810 and 870 nm, 870 and 970 nm, as well as 795 and 970 nm are applied with 2.5 µs interval timing. The wavelengths for the four pairs were chosen with regard to a prevalence of Fd, P700, and PC absorbance changes in the three difference signals 785–840 nm, 810–870 nm, and 870–970 nm, respectively (for information on in vitro and in vivo difference spectra of Fd, P700, and PC see Supplementary Figs. 1, 2, 3, 4). Hence, these difference signals are also referred to as ‘Fd’, ‘P700’, and ‘PC’, although each signal also contains more or less of the respective other two signals. The fourth pair, 795–970 nm, is also referred to as ‘All’, as it is composed of maximal differential changes of all three components over the whole covered NIR range. As will be outlined below (see text describing Figs. 4, 5), the central wavelengths of the four dual-wavelength signals (cw_1_ = 812.5 nm, cw_2_ = 840 nm, cw_3_ = 920 nm, and cw_4_ = 882.5 nm) serve for presenting the signal amplitudes in the form of ‘differential spectral plots’.

Special preamplifier circuitry is employed for mutual compensation of the two signals of each pair (Klughammer and Schreiber 1998). The resulting difference signals are zeroed with the help of an automated balancing routine employing appropriate adjustment of the NIR-ML pulse currents. After balance of the two signals within each of the four pairs, the difference signals can be strongly amplified without risk of amplifier saturation which occurs above 4 V. In this way, differential changes of Δ*I*/*I* in the mV range can be analyzed while the *I* values of the original pulse signals may amount to several hundred V. The latter, however, should be considered just theoretical, as due to effective mutual compensation at no point within the circuitry are high voltage signals generated. This approach not only favors a high signal/noise ratio, but also minimizes electrical and optical artifacts, as long as these affect both signals equally (Klughammer and Schreiber 1998).

As the original single-beam pulse signals are already ‘lost’ during preamplification, for obtaining information on total balanced transmission values, *I*(785) = *I*(840), *I*(810) = *I*(870), *I*(870) = *I*(970), and *I*(795) = *I*(970), a dedicated calibration routine has to be applied. This routine involves a defined increment of pulse current in one of the two channels, leading to an equivalent increment of Δ*I*/*I* in the difference signal. After this calibration step, light-induced changes of the four difference signals are plotted in units of Δ*I*/*I* × 10^−3^.

For special applications (see e.g., determination of transmittance difference spectra in Supplementary Fig. 4) it is also possible to operate the system in the single-wavelength mode. For this purpose a low NIR-ML intensity setting must be used and NIR-ML transmission restricted by an appropriate pinhole diaphragm, so that signal amplitudes are limited to values below 4 V.

### Four difference signals and ‘differential plots’

Information on the redox states of Fd, P700, and PC is contained in the four time-dependent difference signals measured at 785–840 nm (corresponding to cw_1_), 810–870 nm (cw_2)_, 870–970 nm (cw_3_), and 795–970 nm (cw_4_), the amplitudes of which are evaluated with respect to a zero baseline. The latter may be defined for the dark state before actinic illumination or any other state in the course of a recording. A recording may consist of up to 10^6^ data points for each of the four difference signals. Time windows with appropriate widths can be manually set, over which the data points are averaged, for definition of the zero baseline level and determination of a set of ∆*I*/*I* values of the four difference signals. Figure 4a shows an example of typical changes of the four difference signals during dark-light induction of an ivy leaf, with definition of the zero baseline (data points preceding onset of illumination) and two sampling periods for data point averaging defined at 1–2 s and 20–25 s (delineated by vertical broken lines).

Although the courses of the four induction curves in Fig. 4a are similar, differences in the ratios of positive/negative transients are apparent, which are mainly due to different contributions of Fd reduction (negative transients, Schreiber et al. 1988; Klughammer and Schreiber 1991; Klughammer 1992) and P700 + PC oxidation (positive transients) to the four difference signals. The amplitude of every ∆I/I data point corresponds to the sum of light-induced redox changes of Fd, P700, and PC relative to the baseline. Likewise, this is also true for the average of data points over any defined period of time. The two time windows defined in the example of Fig. 4a reveal largely different spectral information. While Fd reduction prevails in the first time window set at 1–2 s, the difference signals in the second time window at 20–25 s are dominated by the oxidation of P700 and PC.

Figure 4b shows a graphical presentation of the spectral information contained in the two sets of ∆*I*/*I* amplitudes defined in Fig. 4a. In contrast to single-wavelength measurements, the spectral information obtained by measurements of *difference signals* cannot be presented in the form of conventional spectra, with signal changes plotted against wavelength (see e.g., conventional difference spectra of Fd, P700, and PC in Supplementary Fig. 4), as each measured difference signal is associated with *two* wavelengths. However, for a graphical presentation of the spectral information the amplitudes of the four difference signals can be plotted against the central wavelengths (cw_*i*_) of the four wavelength pairs, as shown in Fig. 4b. Based on the spectral information contained in such ‘differential plots’ the deconvolution is carried out (see section on ‘Deconvolution and fitting’ below). Mere visual inspection already reveals distinct differences between the differential plots for the 1–2 s (diamonds) and 20–25 s (circles) sampling periods, which may be assumed to be dominated by Fd reduction and P700 + PC oxidation, respectively.

### Selective changes and selective differential plots

Each of the four Δ*I*/*I* values in the differential plots of Fig. 4b reflects the sum of Δ*I*/*I* (Fd), Δ*I*/*I* (P700), and Δ*I*/*I* (PC). For deconvolution of these changes quantitative information on the corresponding differential plots of *selective* Fd, P700, and PC changes are required, which contain the essential information on the *ratios* of differential extinction of each of these components for the four wavelength pairs. In practice, this means that conditions must be found, under which (in a recording analogous to that shown in Fig. 4a) all four difference signals reflect redox changes of *one* of the three components only. When this aim is reached, *selective differential plots* are obtained for each of the three components, the normalized form of which we call ‘Differential Model Plots’ (DMP) (see corresponding section below), with the help of which the information of mixed differential changes can be deconvoluted (see section on ‘Deconvolution and fitting’ below).

We have chosen an empirical approach for determination of *selective differential plots* and the DMP of Fd, P700, and PC in intact leaves. Based on general knowledge of photosynthetic reactions, we expect selective redox changes of these three components under the following conditions and for the following reasons:

1. *Ferredoxin* After dark adaptation the reactions downstream Fd are inactive, so that upon illumination by a light pulse reduced Fd accumulates that is slowly reoxidized in the dark. Initially light-induced reduction of the Fd pool will cause oxidation of P700 and PC which ceases when the pool is filled up and electrons arrive from PS II. Full reduction of P700 and PC may be expected briefly after the light pulse, as oxidation via PS I is stopped while reduction via electrons accumulated in the PQ pool continues. Hence, briefly after the light pulse Fd may be expected to coexist with 100 % reduced P700 and PC. In order to assure that PC is fully reduced before start of illumination, the sample is preilluminated by a multiple turnover pulse of light.
2. *P700* In the presence of FR background light the electron transport chain is depleted of electrons, so that Fd, PC, and P700 may be expected to be largely oxidized. When in this situation a 5-µs sub-saturating single-turnover flash is applied, this will cause almost exclusive turnover of PS II. The electrons set free in PS II lead to transient partial reduction of P700, before this becomes reoxidized by the FR background light. The latter inevitably is linked with some Fd reduction which, however, cannot lead to the accumulation of reduced Fd, when a light-activated sample is used. Due to the high equilibrium constant between PC and P700, PC reduction should be negligibly small.
3. *Plastocyanin* When after emptying the electron transport chain by strong FR light simultaneously with FR-off a saturating single-turnover flash is applied, it may be expected that in the dark after the flash P700 becomes rapidly reduced and Fd oxidized. This means that briefly after FR-off these two components assume the same redox state as before illumination. In contrast, there is a lack of electrons for re-reduction of PC, so that selective PC oxidation may be expected for some period of time following FR-off.

These predictions of optimal conditions for measurements of DMP may be confirmed or questioned by an analysis of the experimental data obtained with the new device. After the DMP of Fd, P700, and PC are determined under the conditions outlined above, these DMP can be applied for deconvolution of a variety of light-induced changes and the resulting redox changes of Fd, P700, and PC can be checked for consistency. Any apparent inconsistency can be analyzed and consequently eliminated by appropriately modifying the conditions of DMP measurements. Hence, the DMP presently applied by the user software of the Dual/KLAS-NIR spectrophotometer have *evolved* from a large number of measurements under a variety of conditions. For example, an unexpected cause of inconsistency encountered at an early stage of development in measurements of Fd-DMP was tracked down to a side effect of the NIR-ML acting as an extremely weak PS I light that causes slow oxidation of a fraction of PC at dark times exceeding 2 min in ivy leaves (not shown in the figures). If this effect is ignored, the assumed selective Fd change is ‘contaminated’ by partial PC reduction. This effect is readily prevented by preillumination of the sample with a short multiple turnover pulse of light (10 ms MT) about 1 min before measurement. Any PC oxidized by the NIR-ML during longer dark-acclimation of the sample will be reduced by the MT and sufficient time is given for reversal of any light activation of Fd oxidation induced by the MT.

### Differential model plots, DMP

**Fig. 6:**
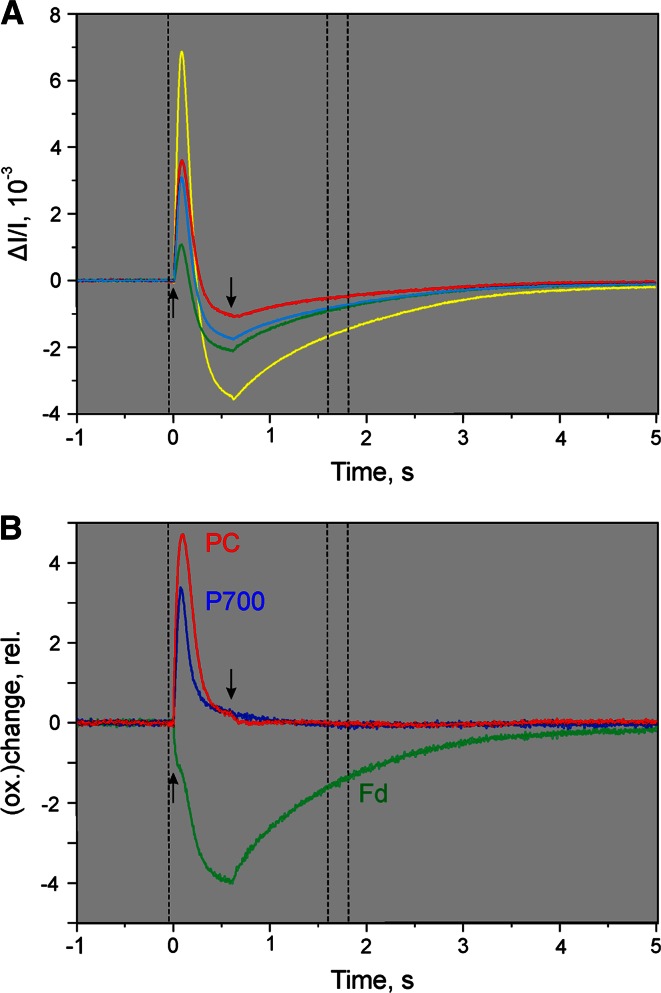
Determination of differential model plot of Fd (Fd-DMP). *Dark-adapted ivy leaf* illuminated for 0.6 s at 300 µmol photons m^−2^s^−1^. 1 min before AL-on a 5-ms MT pulse with 10,000 µmol photons m^−2^s^−1^ was applied. Average of four recordings measured with 10 min repetition rate. *Broken vertical lines* refer to time windows defining zero baseline and Δ*I*/*I* values for DMP. **a** Original recording of difference signals: *Green* 785–840 nm; *yellow* 795–970 nm; *blue* 810–870 nm; *red* 870–970 nm. **b** Kinetics of Fd, P700, and PC deconvoluted from the original kinetics in panel **a**, based on the DMP displayed in Fig. 5. *Upwards and downwards arrows* marking AL-on and AL-off, respectively

**Fig. 7:**
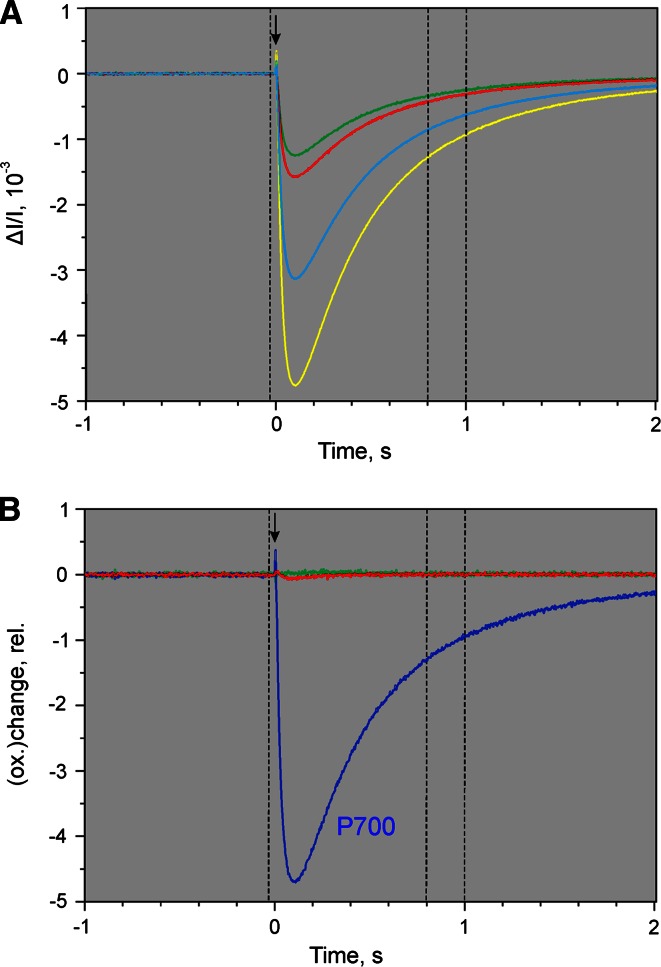
Determination of differential model plot of P700 (P700-DMP). Preilluminated ivy leaf (10 min 630 nm at 300 µmol photons m^−2^s^−1^ plus 3 min dark time plus 3 min 740 nm background light at 40 µmol photons m^−2^s^−1^). Application of 5 µs ST at time 0 (*downward arrow*) in presence of the 740 nm background light. Average of 25 recordings measured with 20 s repetition rate. **a** Original difference signals. **b** Deconvoluted signals. See legend of Fig. 6 for further explanations

**Fig. 8:**
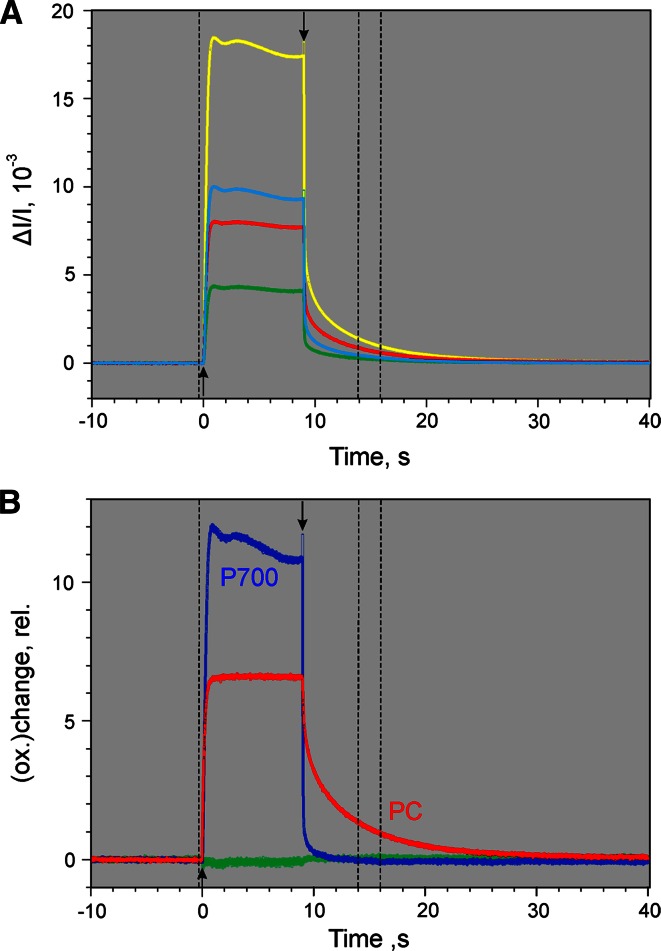
Determination of differential model plot of PC (PC-DMP). Preilluminated ivy leaf (5 min 740 nm at quantum flux density of 360 µmol photons m^−2^s^−1^ plus 30 s dark time). Onset of 9 s FR illumination period with 360 µmol photons m^−2^s^−1^ at time 0 (*upward arrow*). Simultaneously with FR-off application of 50 µs ST (*downward arrow*). Average of four recordings measured with 3 min repetition rate. **a** Original difference signals. **b** Deconvoluted signals. See legend of Fig. 6 for further explanations

The newly developed measuring system enables reliable determination of *normalized selective differential plots* (DMP) for Fd, P700, and PC by pre-programed routines (so-called Triggered Runs) that are optimized for generating selective changes. Such Triggered Run recordings will be presented and discussed in detail below under ‘Results and discussion’ (Figs. 6, 7, 8). Here just the information contained in the resulting selective differential plots and the DMP shall be explained.

Figure 5 shows selective differential plots of Fd, P700, and PC for a dark-green ivy leaf. In Fig. 5a the original four Δ*I*/*I* values of the three components are plotted. As expected, in view of the conditions chosen for obtaining selective changes (see above), Fd and P700 display negative values (reduction) and PC positive values (oxidation). The essential information on the differential extinction coefficients of these three components for the four wavelength pairs is contained in the *ratios* of the four Δ*I*/*I* values, for which it does not matter whether the selective changes are positive or negative. The ratios are also not changed by normalization. In Fig. 5b the selective differential plots are displayed with the maximal values of Δ*I*/*I* being normalized to 1. In this form they serve as Differential model plots, DMP, for deconvolution (see section on ‘Deconvolution and fitting’).

The DMP carry information on the changes of extinction induced in a leaf by selective redox changes of Fd, P700, and PC in the transmittance difference signals of 785–840 nm, 795–970 nm, 810–870 nm, and 870–970 nm. The amplitudes of these changes are determined by the oxidized minus reduced transmittance difference spectra of Fd, P700, and PC in the leaf (see Supplementary Fig. 4). For the purpose of deconvolution of the original four dual-wavelength difference signals the proportions between the four Δ*I*/*I* values of each DMP are decisive, which are directly apparent from the normalized plots. In all the three DMP the 795–970 nm value is maximal (normalized to 1). This can be expected, because bleaching by reduced Fd and absorbance by oxidized P700 and PC decrease over the whole range of wavelengths in the NIR employed by the new device. Hence, the Δ*I*/*I* values at 795–970 nm represent the overall changes (normalized to 1). For comparison, e.g., in the case of the Fd-DMP the ΔI/I value at 785–840 nm amounts to 0.57, which means that 57 % of the overall change of Fd transmittance in the NIR occurs between 785 and 840 nm. In the case of P700, e.g., 67 % of the overall change of P700 transmittance in the NIR occurs between 810 and 870 nm, where the corresponding changes of Fd and PC amount to 49 and 31 % only. In view of such characteristic differences, the DMP constitute specific ‘fingerprints’ of the spectral properties of Fd, P700, and PC in the NIR. Very similar DMP as shown in Fig. 5 were measured in a large variety of leaves, thus suggesting that the spectral properties of Fd, P700, and PC in the NIR are almost independent of leaf species, leaf anatomy, and type of leaf photosynthesis (C3, C4, or CAM metabolism) (Schreiber and Klughammer, in preparation). Comparing the differential plots in Fig. 4b with the three DMP in Fig. 5, it is already clear from visual inspection that the differential plot of the 1–2 s sampling period is dominated by Fd reduction, whereas in the differential plot of the 20–25 s sampling period a mixture of P700 and PC oxidation prevails. The result of deconvolution of all points of the recording will be shown in Fig. 10 below, after introduction of 100 % scaling of redox changes.

**Fig. 9:**
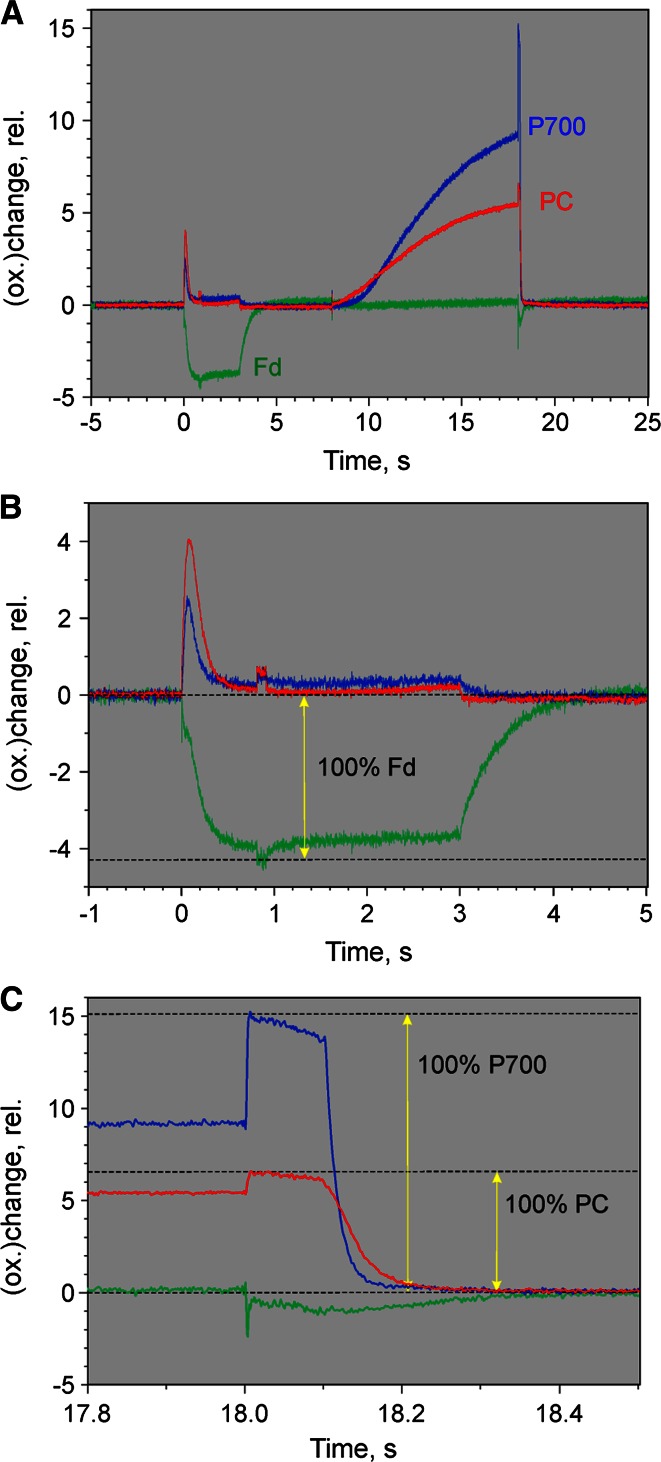
Determination of the relative amplitudes of 100 % redox changes of Fd, P700, and PC in a *dark-green ivy leaf*, which served for scaling of the 100 % DMP values in Table 1. **a** Redox changes induced after dark-adaptation during the course of a pre-programed ‘Triggered Run’ involving a 3-s illumination period with 630 nm AL at 300 µmol photons m^−2^s^−1^ starting at time 0, application of 100 ms MT with 10,000 µmol photons m^−2^s^−1^ at 0.8 and 18 s, as well as 10 s illumination period with 740 nm FR (360 µmol photons m^−2^s^−1^) starting at 8 s. **b** Zoomed detail of recording in panel **a** depicting determination of 100 % Fd change. **c** Zoomed detail of recording in panel **a** depicting determination of 100 % P700 and PC changes

**Fig. 10:**
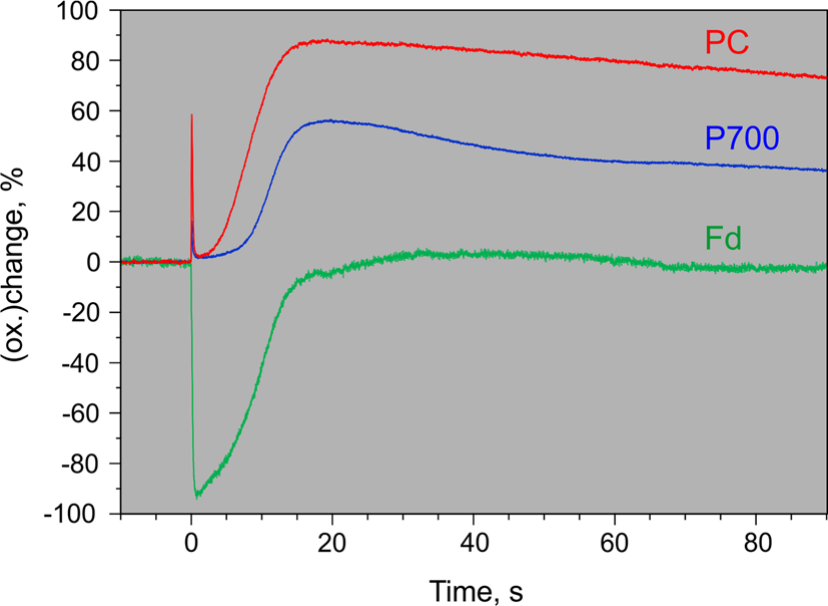
Deconvoluted kinetics of light-induced Fd, P700, and PC changes derived from original recordings of difference signals in Fig. 4, with display of ±100 % redox changes. Deconvolution based on determinations of 100 % changes in Fig. 9 and the derived 100 % DMP values listed in Table 1. For conditions see legend of Fig. 4

As long as no information is available on the effective differential extinction coefficients of Fd, P700, and PC in a given sample, differences of extinction of these components cannot be taken into account and only the *relative* differential changes of each component in the four difference signals are analyzed. Once deconvolution has provided information on the *maximal* changes induced by 100 % redox changes (see below: Fig. 9 under ‘Results and discussion’) the values of Fd-DMS, P700-DMS, and PC-DMS can be normalized to 100 % redox changes (see Table 1 below). When this is done, the amplitudes of the deconvoluted signals directly represent percent oxidation of Fd, P700, and PC (see e.g., Figure 10 below under ‘Results and discussion’ section for the original dark-light induction recording of Fig. 4a).

### Deconvolution and fitting

The four time-dependent difference signals are deconvoluted with the help of a dedicated fitting routine. At any time each of these signals *S*_1_, *S*_2_, *S*_3_, and *S*_4_ (not to be confused with *S*-states of the oxygen evolving complex) consists of the sum of the DMP values for P700, PC, and Fd at the central wavelengths, cw_*i*_, multiplied by the contributing amplitudes $a_{\text{P700}} , \;a_{\text{PC}} , \;a_{\text{Fd}}$ respectively:

1 $$S_{i} = a_{\text{P700}} \cdot {\text{DMP}}_{\text{P700}} \left( {{\text{cw}}_{i} } \right) + a_{\text{PC}} \cdot {\text{DMP}}_{\text{PC}} \left( {{\text{cw}}_{i} } \right) + a_{\text{Fd}} \cdot {\text{DMP}}_{\text{Fd}} \left( {{\text{cw}}_{i} } \right);\quad i = 1,2,3,4$$

Changes of the amplitudes $a_{\text{P700}} , \;a_{\text{PC}} , \;a_{\text{Fd}}$ are directly proportional to changes of the redox states of P700, PC, and Fd; they are calculated by solving the above linear equation system (Eq. 1) with the help of a multilinear regression procedure using singular value decomposition. This procedure generates the exact solution for four linear independent components in the case of the fully determined equation system. Here, when only amplitudes of the three components are calculated, the above equation system is over determined. In this case the regression procedure calculates the best approximated solution by minimizing the deviations. After calculating the amplitudes for every point in time, the time course of P700, PC, and Fd redox changes together with Chl *a* fluorescence changes are plotted. Although mathematically three independent signals would have been sufficient for the deconvolution of these three components, the fourth signal provides an additional degree of freedom, thus lowering the interference of any disturbing non-specific signal changes. Up to a time resolution of 1 ms/point deconvolution and fitting can be performed in real time, so that the time-dependent changes of the three deconvoluted signals can be directly followed on the monitor screen.

### Automated software routines

**Fig. 11:**
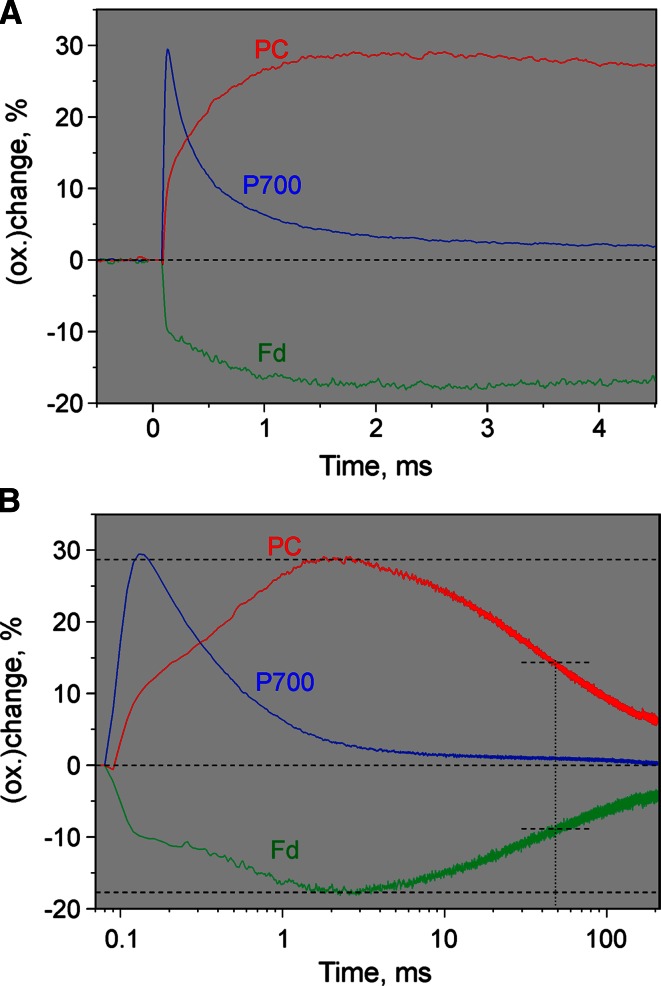
Redox changes of Fd, P700, and PC induced by a non-saturating 20 µs flash in a dark-adapted ivy leaf. Application of special Script routine for repetitive measurements of individual dual-wavelength difference signals at maximal time resolution (see ‘Materials and methods’). 100 recordings of each difference signal were averaged before deconvolution. **a** Linear time scale. **b** Logarithmic time scale. Maximal levels of PC oxidation and Fd reduction as well as 50 % re-reduction of PC and reoxidation of Fd are indicated

**Fig. 12:**
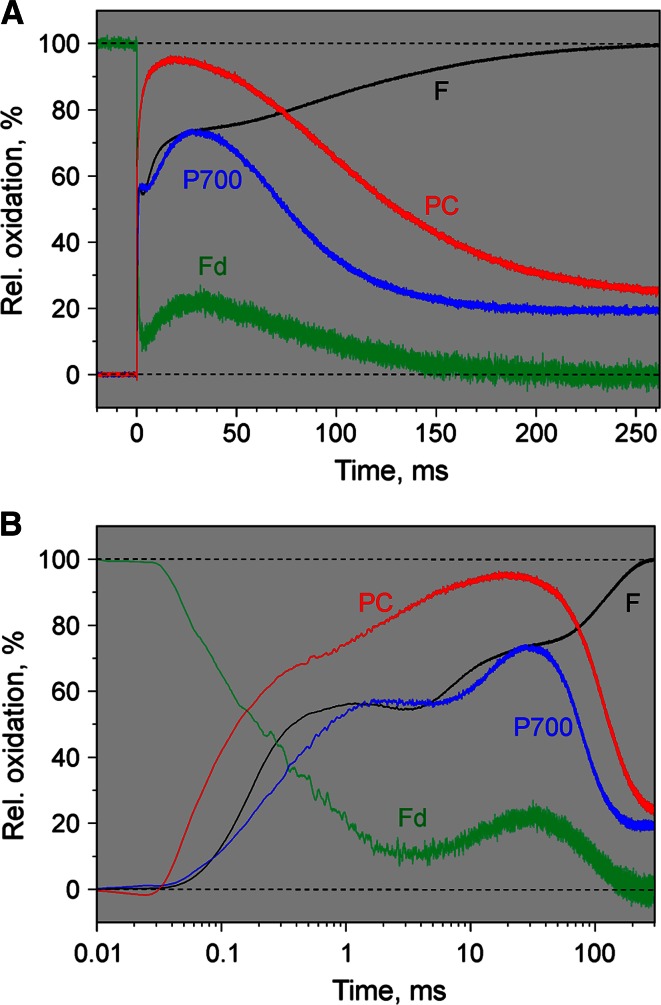
Comparison of polyphasic fluorescence rise and deconvoluted redox changes of Fd, P700, and PC induced upon illumination of *dark-adapted ivy leaf* with a strong 300 ms MT pulse (15,000 µmol photons m^−2^s^−1^ 630 nm). Repetitive measurements of individual dual-wavelength signals and fluorescence signal at maximal time resolution with 3 min dark times between MT applications. Averages of 23 recordings of each difference signal and fluorescence. **a** Linear time scale. **b** Logarithmic time scale. *F*, variable fluorescence, with 0 % signal corresponding to minimal fluorescence yield Fo (O-level) and 100 % to maximal yield Fm (P-level); the amplitude reached at the end of the photochemical phase at about 1 ms corresponds to the *I*_1_-level (Schreiber 1986) or J-level (Strasser et al. 1995) and the amplitude reached at the end of the first thermal phase at 30–50 ms to the *I*_2_- or *I*-level, respectively

The new device is operated via a dedicated software supporting numerous automated routines which decisively contribute to the reliability and reproducibility of data acquisition using the new device. Besides automated calibration and *on*-*line* deconvolution (outlined above) the most important of these routines are:

- *Triggered Runs* for reproduction of a sequence of trigger events (including AL-on/off, FR-on/off, ST, MT) using a defined time sequence with ms resolution. The recordings of Figs. 6, 7, 8, 9 are based on Triggered Runs (saved in so-called STM-files) that can be reproducibly and reliably applied even by non-experts.
- *Fast trigger files* for reproduction of sequences of trigger events timed with 2.5 µs resolution, including switching of pulse frequency and preamplifier gating. The recordings of Figs. 11 and 12 were carried out using Fast trigger files (FTM-files).
- *Averaging* for improving the signal/noise ratio of recordings measured repetitively with defined clock intervals using STM- and FTM-files or Scripts.
- *Scripts* for reproduction of a sequence of trigger events timed with 1 s resolution, including start of STM- and FTM-files as well as averaging of the obtained recordings. All commands that can be given manually can also be programed in Scripts. The recordings of Figs. 11 and 12 were carried out using a special Script programed for measurements at maximal time resolution, involving repetitive cycling between the four dual-wavelength and fluorescence channels, triggering of FTM-files at pre-programed repetition rates and averaging of recordings.
- *Baseline correction* for compensation of unavoidable baseline drifts in the original dual-wavelength recordings. For this purpose the pre-trigger baseline drift is determined and subtracted from the recording.

### Plant material

All measurements were carried out with detached dark-green ivy leaves (*Hedera helix*), the petioles of which were kept in water. The leaves were harvested from a balcony exposed to natural daylight, with daily full exposure to sun light for about 6 h during August 2015, when measurements were carried out.

## Results and discussion

### Determination of differential model plots and deconvolution of selective Fd, P700, and PC changes

As outlined under ‘Materials and methods’ (see Fig. 4 and sections on ‘Selective changes and selective differential plots’ and ‘Differential model plots, DMP’), deconvolution of the four differential transmittance changes (785–840 nm, 810–870 nm, 870–970 nm, and 795–970 nm) into Fd, P700, and PC redox changes requires spectral information on *selective* changes induced in each of these three components. Based on this information Differential model plots (DMP) for these components can be derived, with the help of which the overall differential changes can be deconvoluted. The three DMP for *Hedera helix* were already shown in Fig. 5b (‘Materials and methods’), representative for selective changes of Fd, P700, and PC. In the following Figs. 6, 7, 8 the *original experimental data* are presented, from which these three DMP were derived. The design of the underlying experiments is based on common knowledge of photosynthetic electron transport and experience gained in the course of more than 1 year of practical applications of the new device (see section on ‘Selective changes and selective differential plots’). The applied instrument settings and illumination programs are stored in dedicated ‘Scripts’ and ‘Trigger files’ which can be used to start automated illumination programs, so-called ‘Triggered Runs’, at defined repetition rates with the help of a ‘clock’. A number of Triggered Run recordings can be averaged to obtain the desired signal/noise ratio. The following presentation of results in Figs. 6, 7, 8 serves two purposes. First, to show the original data on selective changes of Fd, P700, and PC, from which the DMP of Fd, P700, and PC in Fig. 5 were derived. Second, to present the deconvoluted data and to demonstrate the consistency of these data with the assumptions made for definitions of conditions to assure selective changes of the three components (see ‘Selective changes and selective differential plots’).

Figure 6a shows the original differential changes measured for the assessment of a *selective* Fd change. For obtaining such information it is *not* necessary that *all* data points of the measured changes are selective for Fd. As explained above (see section on ‘Selective changes and selective differential plots’) selectivity needs to be fulfilled during the sampling period defined by the two broken vertical lines with respect to the zero baseline only. Also it is *not* required that a 100 % redox change is induced. What counts are the ratios between the amplitudes of the four difference signals. The information on absolute amplitudes will be lost anyways upon normalization of the amplitude values (see Fig. 5).

In the example of Fig. 6a, a dark-adapted ivy leaf was illuminated for 0.6 s at moderate actinic intensity (300 µE m^−2^ s^−1^) and the resulting dark-light–dark transients of the four difference signals were recorded. The zero baselines were defined by the signals recorded during the 1 s period preceding AL-on. The sampling time period during which a selective Fd signal is assumed, is defined by the two broken vertical lines (200 ms period starting 800 ms after AL-off). Four recordings triggered with 10 min clock intervals were averaged.

The rationale for the experiment depicted in Fig. 6a is that after dark-acclimation the enzymatic reactions at the PS I acceptor side are largely slowed down, so that the primary PS I acceptor pool, consisting mainly of Fd, is readily reduced upon illumination. Oxidation of P700 and PC accompanying Fd reduction is transient only, due to rapid re-reduction by electrons arriving from PS II and the fact that the acceptor pool becomes exhausted. PS I turnover stops

instantly upon AL-off and any remaining oxidized PC and P700 will be quickly reduced by electrons accumulated during actinic illumination in the intersystem chain. On the contrary, under the given conditions Fd reoxidation should be rather slow, so that an appreciable amount of Fd will remain reduced at 1 s after AL-off. Hence, during the 0.8–1 s time period after AL-off all four differential signals should just reflect the reduction of Fd with respect to the dark state. Based on the averaged values of the four signals during this time period the Fd-DMP depicted in Fig. 5 (green trace) was derived. The original values were normalized (maximal value of Δ*I*/*I* (795–970 nm) = 1).

Figure 6b shows the Fd, P700, and PC changes deconvoluted from the original recordings in Fig. 6a with the help of the DMP depicted in Fig. 5. While the changes of the four original signals are quite similar, the deconvoluted changes of Fd differ considerably from those of P700 and PC. A rapid phase of Fd *reduction* is paralleled by rapid *oxidation* of P700 and PC. A secondary slower phase of Fd *reduction* kinetically correlates with P700 and PC *re*-*reduction*. Antiparallel changes of Fd and PS I donors may be expected upon start of illumination, before arrival of electrons from PS II. Thereafter Fd reduction continues while P700 and PC approach complete reduction. After AL-off the PC and P700 signals rapidly reach the zero baseline (full reduction), whereas Fd reoxidation is rather slow (*t*_1/2_ = 0.8 s). At 0.8–1 s after AL-off (indicated by the broken vertical lines) Fd reduction still is appreciable. The deconvoluted data show a wealth of specific information on the dark-light induction characteristics of Fd, P700, and PC, which appear consistent with the present state of knowledge on primary photosynthetic reactions.

The original dual-wavelength difference recordings for determination of the P700-DMP (via a selective redox change of P700) are shown in Fig. 7a. The same ivy leaf as in the experiment of Fig. 6 was used. In Fig. 7b the corresponding deconvoluted changes of P700, PC, and Fd are presented. A light-adapted ivy leaf (10 min at 300 µmol photons m^−2^s^−1^) was pre-darkened for 3 min and then preilluminated by weak intensity FR light until all the four

signals reached stationary levels. Then a Triggered Run recording was started, the baseline recorded (in the presence of FR background light) and 3 s after start of the recording, a 5-µs single-turnover flash (ST) was applied. The ST-induced changes of the four original signals were similar: Small positive spikes were followed by larger negative changes that slowly relaxed (*t*_1/2_ = 500 ms) towards the baseline. The time interval during which data points were averaged for P700-DMP determination were placed between 0.9 and 1 s after ST application (broken vertical lines).

The deconvoluted changes in Fig. 7b suggest that the conditions, under which the original changes in Fig. 7a were recorded, were properly chosen to induce selective P700 changes: In the presence of weak FR background light PC and Fd can be expected to be almost completely oxidized and P700 largely oxidized. The 5-µs ST induces a single turnover in the small fraction of non-oxidized P700 thus causing some oxidation of P700 which, however, will be quickly re-reduced by electrons arriving from ST-induced PS II turnover. While oxidation of a small fraction of P700 should lead to reduction of a correspondingly small fraction of Fd, the latter change may be too small to be detected, particularly as a light-activated sample was used, so that reoxidation of reduced Fd will be relatively fast. In contrast, reoxidation of PS II-reduced P700 by the weak continuous FR background light is relatively slow. On their way from PS II to PS I, the electrons may cause some transient PC reduction which, however, should be negligibly small due to the distinctly higher oxidation potential of P700. All these aspects are in line with the assumption that the changes induced by a 5-µs ST in the presence of FR are primarily due to P700 and selectively reflecting P700 at 1 s after application of the ST. As expected, the deconvoluted data in Fig. 7b reveal practically exclusive changes of P700.

Figure 8a shows the original dual-wavelength recordings for the determination of PC-DMP. For comparison, in Fig. 8b the corresponding deconvoluted changes are presented. The same ivy leaf as used in the experiments of Figs. 6 and 7 was first illuminated with relatively strong FR in order to oxidize P700, PC, and the intersystem electron transport chain. Simultaneously with FR-off a saturating 50 µs ST was applied, so that a single electron per chain was sent from PS II towards PS I. With P700 displaying a much higher oxidation potential, its re-reduction will be much faster than that of PC. While the relatively strong FR and the final ST may be expected to cause some Fd reduction, complete reoxidation of any such reduced Fd should occur within a couple of seconds. Hence, it appears safe to assume that the signal changes observed 5–7 s after FR-off (as defined by the broken vertical lines) selectively reflect PC. This assessment is confirmed by the deconvoluted data in panel B.

We note that irrespective of whether the DMP for a particular component is correct or not, the deconvolution based on this DMP will always calculate zero for the two other components *within the time window* for which a selective change was assumed. Therefore, the most important criterion for proper choice of the reference time window is that after deconvolution the two other signals do not show any significant changes *shortly before and after* the reference window. This criterion is fulfilled for the above determinations of the DMP of Fd, P700, and PC.

### Assessment of maximal differential transmittance changes

In the DMP depicted in Fig. 5 the largest changes of all the three components occur in the 795–970 nm signals (‘All’, Central Wavelength 882.5 nm), which are normalized to 1. This means that for the time being, as long as no information on the differential extinction coefficients of Fd, P700, and PC is available for the four difference signals in the given leaf sample, the deconvoluted data just describe *relative* changes of the redox states of the three components. Consequently, the deconvoluted changes shown in panel B of Figs. 6, 7, and 8 were presented in *relative* units of oxidation. For information on the *absolute* extents of redox changes in units of % oxidation, the *maximal* differential transmittance changes associated with 100 % redox changes of the three components have to be known.

In the experiments of Figs. 6, 7, and 8 the measured differential transmittance changes were intended to be *selective*, but *not maximal*. Actually, in practice it is not possible to induce a *maximal* change of one of the three redox signals without changing at the same time the two other signals. The information on maximal changes has to be obtained by separate measurements, as described below.

One-hundred percent redox changes of Fd, P700, and PC can be induced with the help of a single appropriately programed Triggered Run recording, as shown in Fig. 9 (A–C). The displayed signals were deconvoluted using the DMP in Fig. 5. The same dark-adapted leaf, as also used in the experiments of Figs. 4, 6, 7, and 8, was first illuminated with AL at moderate intensity (300 µmol photons m^−2^s^−1^ 630 nm) until within a second a stationary level of Fd reduction was reached. Then in addition a saturating 100 ms multiple turnover light pulse (MT) was applied.

We may assume that Fd becomes fully reduced during the MT, as under the given conditions the rate of Fd reoxidation is low (see Fig. 6b). Along with 100 % Fd reduction also some P700 and PC oxidation occurs which, however, in this case is of no concern, as it does not prevent full reduction of Fd and is accounted for by deconvolution. Panel 9B shows the zoomed part of the recording relating to 100 % Fd determination, which amounts to 4.3 arbitrary units of redox change.

In the same Triggered Run, following the initial actinic illumination with 630 nm light that drives both photosystems, the leaf was illuminated for 10 s with strong 740 nm light (FR) that almost selectively drives PS I, thus largely oxidizing PC and P700. Simultaneously with FR-off another saturating 100 ms MT was applied. The action of this MT is twofold: First, it causes 100 % oxidation of PC and P700 by PS I activity; second, it assures by PS II activity that P700 and PC rapidly become 100 % reduced in the following dark period. The electrons set free in PS II reach the PS I donors with a delay of 5–10 ms, so that the time interval during which maximal oxidation of P700 and PC can be observed is rather short and determination of the maximal amplitude requires relatively high time resolution and extrapolation to 5 ms (Klughammer and Schreiber 1994). Panel C of Fig. 9 shows the zoomed part of the recording relating to 100 % P700 and 100 % PC determinations. A 100 % change of P700 and of PC corresponds to 15.1 and 6.6 arbitrary units, respectively, as compared to the 4.3 arbitrary units for a 100 % Fd change.

Obviously, with the given sample and measuring system, a 100 % P700 redox change is detected by a factor of 15.1/6.6 = 2.3 more sensitive than a 100 % PC redox change and by a factor of 15.1/4.3 = 3.5 more sensitive than a 100 % Fd redox change. The differences in ‘sensitivity’ are mainly due to differences in the effective in vivo differential extinction coefficients of Fd, P700, and PC for the four wavelength pairs. In view of Fd/P700 and PC/P700 ratios of 4.5 and 3.0, respectively, in spinach (Klughammer 1992) it may be estimated that the molar extinction coefficient of P700 in the NIR is roughly 16 times higher than that of Fd and 7 times higher than that of PC. These estimates illustrate the technical challenge involved in the deconvolution of Fd and PC redox changes from those of P700.

The values of maximal changes of the three deconvoluted signals (4.3, 15.1, and 6.6 arbitrary units for Fd, P700, and PC, respectively) can be applied for re-scaling the DMP values of Fig. 5 such that the deconvoluted signals of the three components will be displayed on a % redox change scale. The scaling does *not* affect the deconvolution as such, which solely relies on the proportion of the four values *within* a given DMP. Scaling simply changes the proportions *between* the three DMP. In Table 1 the original DMP values (with *all* maximal values normalized to 1.000, as plotted in Fig. 5) and the 100 % DMP values are listed. 

Figure 10 shows the deconvolution of the original recording presented in Fig. 4a making use of the 100 % DMP values displayed in Table 1. It is apparent that dark-light induction initially (i.e., during the first 10 s) is dominated by Fd reduction which reaches almost 100 %, whereas P700 and PC after initial oxidation spikes remain close to fully reduced during the first 10 s of illumination. During 10–30 s after AL-on the reoxidation of Fd is first paralleled by PC oxidation. Significant P700 oxidation does not set in before about 15 s after AL-on where PC already is 50 % oxidized. Towards the end of the recording close to stationary redox states are reached, characterized by full oxidation of Fd, 30 % oxidation of P700, and 70 % oxidation of PC. This means that under the given conditions, with continuous illumination at moderate intensity (300 µmol photons m^−2^ s^−1^ red light) PS I quantum yield is limited by the donor side. Whereas PS I units containing P700^+^ cannot contribute to photochemical energy conversion, accumulation of PC^+^ does not affect the PS I quantum yield. Therefore, without deconvolution the PS I quantum yield would be underestimated. For example, evaluation of the original 810–870 nm difference signal in Fig. 4 (before deconvolution) results in a stationary PS I quantum yield of 0.58, as compared to a value of 0.70 after deconvolution based on all four original signals.

**Table 1:** DMP values of *Hedera helix* derived from selective changes of P700, PC, and Fd (maximal changes normalized to 1.000) (see Figs. 5, 6, 7, 8) and after scaling according to 100 % redox changes using the scaling factors 4.3/100, 15.1/100, and 6.7/100 for Fd, P700, and PC, respectively (as derived from the data in Fig. 9)

Component	∆*I*/*I*(785–840) 812.5 nm		∆*I*/*I*(810–870) 840 nm		∆*I*/(795–970) 882.5 nm		∆*I*/*I*(870–970) 920 nm	
	norm.	100 %	norm.	100 %	norm.	100 %	norm.	100 %
Fd	0.5560	0.0239	0.4979	0.0214	1.0000	0.0430	0.3182	0.0137
P700	0.2692	0.0406	0.6709	0.1013	1.0000	0.1510	0.3363	0.0508
PC	0.1896	0.0125	0.3221	0.0213	1.0000	0.0660	0.6308	0.0416

Once the 100 % DMP values for a particular sample are known and saved in the software, deconvolution of light-induced Fd, P700, and PC changes as depicted in Fig. 10 can be performed *on*-*line* in real time.

### Deconvolution of flash-induced sub-ms kinetics at maximal time resolution

The standard time resolution of the new device is about 1 ms (100 µs per data point) which is sufficient for all standard applications, including determination of MT-induced maximal levels of Fd reduction, P700 oxidation, and PC oxidation, as demonstrated in Fig. 9b, c, respectively. Time resolution is limited by the fact that the four dual-wavelength difference signals and up to two fluorescence signals are measured *quasi*-*simultaneously* by rapid sequential alteration of the ten different types of ML at kHz rate. A distinct advantage of this approach of quasi-simultaneousness is the fact that the sample is ‘seen’ by all signals in the *same state*. This cannot always be assured by other types of kinetic spectrophotometers when, e.g., for each wavelength of ML a filter has to be changed (Joliot et al. 2004). There are, however, applications where it is easy to keep a defined state stable over a long time, as, e.g., in the case of the dark state or of a steady state maintained by illumination. In such applications, the new device offers the possibility to measure the four dual-wavelengths difference signals (or fluorescence signals) one after the other separately at maximal time resolution of about 30 µs (2.5 µs/data point). In this way, and in conjunction with averaging of a number of recordings, maximal time resolution can be combined with high signal/noise ratio (see ‘Automated software routines’ under ‘Materials and methods’ section).

An example of a Fast Kinetics measurement at maximal time resolution is given in Fig. 11 which shows the deconvoluted changes of Fd, P700, and PC in a dark-adapted ivy leaf induced by a non-saturating 20 µs flash. Notably both Fd and PC display distinct *biphasic* responses of flash-induced reduction and oxidation, respectively. The redox changes were deconvoluted using 100 % DMP scaling (see Table 1; Figs. 9, 10) so that the amplitudes reflect percent of maximal redox changes. In the case of Fd, overall reduction induced by the 20 µs flash amounts to about 17 %, with the rapid phase being larger than the slow one. While the first phase is too fast to be time resolved, the second phase displays a half-time of about 0.6 ms. The half-time of Fd reoxidation, estimated from Fig. 11b, amounts to 50 ms.

For understanding the biphasic kinetics of flash-induced Fd reduction, it has to be considered that a 20-µs flash allows more than one turnover in PS I (Haehnel 1984). Therefore, it appears likely that the two phases are related to the two fractions of PS I reaction centers with one or two turnovers. While the 1st turnover may be expected to result in fast reduction of Fd bound to PS I (Sétif 2001), the 2nd turnover cannot lead to Fd reduction before the Fd reduced by the 1st turnover is released from its binding site and an oxidized Fd has bound to the complex. This interpretation should be considered tentative until information on possible contributions of the [4Fe–4S] clusters to the NIR signals are available. As already pointed out in the ‘Introduction’ section, the spectra of *F*_X_, *F*_A_, and *F*_B_ redox changes in the NIR presently are not yet known. Therefore, for the time being the above explanation of the data in Fig. 11 is hypothetical, requiring confirmation by future experiments.

The electrons reducing Fd ultimately are derived from water, but through PC and then P700. The amplitude of overall P700 oxidation by the 20 µs flash is diminished by its fast re-reduction already *during* the 20 µs flash by PC bound to the PS I reaction center complex (Haehnel 1984; Drepper et al. 1996). Hence, the observed response of P700^+^ should be mostly caused by the 2nd turnover. The ensuing re-reduction of P700^+^ is biphasic, the dominating first phase of which displays a half-time of about 350 µs. Two competing sources of electrons for P700^+^ re-reduction can be envisaged: The reduced terminal FeS cluster, (*F*_A_*F*_B_)^−^ (recombination), and PC.

As already argued above, the rapid phase of PC oxidation may be assumed to parallel re-reduction of P700^+^ after the 1st turnover (first-order rate constant of 11 µs), which is too fast to be resolved by our set-up. The slow phase displays a half-life of close to 400 µs, which is similar to the *observed* half-time of P700^+^ re-reduction. Hence, under the given condition recombination does not seem to play any significant role and the P700^+^ re-reduction kinetics seem to be dominated by the bimolecular reaction between soluble PC and PS I (Drepper et al. 1996).

The re-reduction kinetics of PC (Fig. 11b), which are much slower than those of P700, are almost identical to the reoxidation kinetics of Fd, displaying the same half-time of ca. 50 ms. This observation agrees with the interpretation that flash-oxidized P700 is rapidly re-reduced by PC and by the ‘linear electron’ originating from turnover of PS II, whereas under the given conditions flash-oxidized PC is slowly re-reduced by ‘cyclic electrons’ originating from the flash-reduced Fd. More extended measurements will be required to support this interpretation. If proven correct, the data presented in Fig. 11b would be the first *direct* measurement of Fd-dependent cyclic electron flow in vivo.

### Parallel measurements of chlorophyll fluorescence

With the new device Chl *a* fluorescence is treated as just one out of five NIR signals (see ‘Materials and methods’). Similar to the four NIR-transmittance difference signals, it represents a ‘whole leaf signal’ as green ML (540 nm) is applied and long-wavelength emission (>720 nm) is measured. Hence, the derived information on PS I and PS II properties are directly comparable. An example of parallel measurements of Chl *a* fluorescence and deconvoluted Fd, P700, and PC changes is presented in Fig. 12. Measurements were carried out at maximal time resolution, as already outlined for the experiment of Fig. 11.

In the experiment of Fig. 12 the leaf was dark-adapted for 30 min and then exposed to a saturating multiple turnover light pulse, MT. The responses of Fd, P700, PC, and Chl fluorescence, F, are displayed on a linear time scale (panel A) and on a logarithmic time scale (panel B). The *F* induction kinetics correspond to the well-known ‘polyphasic fluorescence rise’ (Schreiber 1986; Neubauer and Schreiber 1987; Schreiber and Neubauer 1987; Strasser et al. 1995), with the so-called ‘photochemical phase’ (up to ~1 ms) preceding two well-separated ‘thermal phases’ (see review by Stirbet and Govindjee 2012). The following kinetic correlations and features are apparent:

- the photochemical phase of fluorescence induction kinetically correlates with rapid phases of P700 and PC oxidation paralleled by Fd reduction, which reaches ~90 % within about 3 ms;
- rapid phases of PC and P700 oxidation in the sub-ms range are followed by well-separated slower phases at 1–20 ms in the case of PC and 8–20 ms in the case of P700;
- oxidation of PC precedes that of P700 in both phases;
- in the given dark-adapted state MT-induced oxidation of P700 reaches about 55 % in the first and 73 % in the second step, as compared to about 72 and 95 % oxidation in the case of PC;
- the first thermal phase of fluorescence induction is paralleled by Fd *reoxidation*, whereas the second thermal phase leading to maximal fluorescence yield, Fm, kinetically correlates with an increase of Fd *reduction* to 100 %;

Previous work comparing the induction kinetics of Chl fluorescence and 820–830 nm transmittance changes upon onset of strong actinic illumination (Schreiber et al. 1989; Schansker et al. 2003), in which it was assumed that light-induced changes of 820–830 nm transmittance are representative of P700 redox changes, had already suggested that P700^+^ re-reduction during the multiple turnover pulse of strong light correlates with the second thermal phase of fluorescence induction. Not unexpectedly, the deconvoluted data of Fig. 12 show that these changes are paralleled with the final 100 % reduction of the Fd pool. An unexpected finding is the kinetic correlation between the first thermal phase of variable fluorescence and an intermediate phase of Fd *reoxidation*. Notably, during the same time interval PC and P700 become further oxidized. In principal, such unexpected behavior could be explained by a backflow of electrons from reduced Fd to plastoquinone at the acceptor side of PS II. However, other explanations may be possible. While it is clear that such data of parallel measurements of fluorescence, Fd, P700, and PC reveal a wealth of important information on the interplay of PS II with acceptor and donor sides of PS I, which deserve future attention, further discussion within the framework of the present technical report would be out of the scope of this report.

## Summary, conclusions and outlook

The presented data demonstrate that it is possible to deconvolute Fd, P700, and PC redox changes in intact leaves without knowledge of the effective sample- and wavelength-dependent extinction coefficients of these components. For this purpose we have developed a dedicated pulse-modulated measuring system for four simultaneous dual-wavelength difference signals in the 785–970 nm spectral range (see Figs. 1, 2, 3, 4) and have conceived an *empirical* approach for reliable deconvolution of these signals. Central to this approach are standard measurements of light-induced kinetic changes carried out under particular conditions that allow one to define time windows during which *selective* changes of a single component may be assumed (see Figs. 6, 7, 8). Based on this information, so-called Differential model plots (DMP) for the three components were derived (Fig. 5; Table 1). The DMP values are essential for deconvoluting the amplitudes of the contributions of the three components by an automated routine that solves a simple linear equations system outlined under ‘Materials and methods’ section (Eq. 1). Thanks to high processor rates of modern computers, deconvolution can take place *on*-*line*, i.e., in real time during the course of a recording with up to 1 kHz time resolution. For still higher time resolution, use of a special routine for ‘cyclic averaging’ of individual dual-wavelength difference signal changes can be made, e.g., for studying flash relaxation kinetics (Fig. 11) or the fast induction transients paralleling the polyphasic fluorescence rise upon onset of saturating light (Fig. 12). Using a pre-programed, automated ‘Triggered Run’ the amplitudes of 100 % redox changes of Fd or P700 and PC are readily determined and can be applied for scaling deconvoluted signals in percent of total reduction or oxidation, respectively (Fig. 9 and examples in Figs. 10, 11, 12).

The new measuring system essentially provides three major lines of novel information:

1. *On the ‘openness’ of the PS I acceptor side and on the soluble pool of Fd in particular.* As already stated in the ‘Introduction’ section, we cannot exclude the possibility that besides the [2Fe–2S] cluster of soluble Fd also the core-bound [4Fe–4S] clusters *F*_X_, *F*_A_, and *F*_B_ may contribute to the ‘Fd’ signal. For clarification of this aspect in vitro measurements with the new device using PS I particles are required. As the redox potential of Fd is distinctly higher than that of the core FeS clusters, it may be expected that under normal physiological conditions electrons will preferentially end up in the soluble Fd pool from where they are distributed to the various enzymatic pathways. Electron transfer reactions following the binding of reduced Fd to numerous stromal enzymes have been the topic of a vast number of mainly molecular biological studies (for a recent review see Hanke and Mulo 2013). So far no tool has been available to monitor the changes of the Fd redox state in vivo. As the time resolution of the new device allows us to analyze flash relaxation kinetics with high accuracy, it may be foreseen that important new in vivo information may be obtained by studying the re-reduction kinetics following a strong pulse of light as a function of the numerous parameters modulating the activity of the various Fd-dependent pathways.
2. *Redox state of P700 and quantum yield of PS I.* The new device allows, for the first time, accurate on-line determination of P700^+^ in intact leaves, without any assumptions on relative effective extinction coefficients of P700^+^/PC^+^ and on the equilibrium between the P700 and PC redox couples. Reliable detection of P700^+^ signals (without distortion by potential PC and Fd changes) is particularly important for analysis of post-illumination re-reduction kinetics which in the past have played a crucial role in estimating cyclic PS I electron transport (CET). Furthermore, reliable differentiation between P700^+^ and PC^+^ is a prerequisite for a thorough investigation of heterogeneous properties of P700 and PC, resulting from the location of PS I in different membrane regions (Albertsson 2001). Reliable deconvolution of P700^+^ is also essential for assessment of PS I quantum yield which may be substantially underestimated when not differentiated from PC^+^ (see also text accompanying Fig. 10).
3. *Redox state of PC.* While in the past only a few specialized laboratories have attempted to deconvolute PC from P700 redox changes, the new device now enables reliable on-line deconvolution of these two components. Details of PC binding to PS I and the redox equilibration between PC and P700 have been studied intensively in vitro (Drepper et al. 1996), whereas not much details is known about the reactions in vivo. Schöttler et al. (2004) demonstrated large changes in PC/P700 ratios parallel to changes in photosynthetic capacity (see also recent review of Schöttler and Toth 2014). The new device should become an important tool for routine determinations of PC/P700 that might qualify as a convenient proxy of photosynthetic capacity.

## Electronic supplementary material

Below is the link to the electronic supplementary material.

Supplementary material 1 (PDF 378 kb)

## Abbreviations

AL: Actinic light
CET: Cyclic electron transport in photosystem I (PS I)
COB: Chip-on-board array of light emitting diodes
DMP: Differential model plot
Fd: Ferredoxin
*F*_X_, *F*_A_, *F*_B_: Iron sulfur clusters on the acceptor side of PS I core complex
FNR: Ferredoxin-NADP Reductase
FR: Far-red light
*I*/*I*_0_: Ratio of transmitted/incident light (transmittance)
LED: Light emitting diode
ML: Pulse-modulated measuring light
MAP: Mehler–ascorbate peroxidase cycle
MT: Multiple turnover light pulse
NADP: Nicotinamide adenine dinucleotide phosphate
NIR: Near-infrared light
PAM: Pulse-amplitude modulation
PC: Plastocyanin
P700: Reaction center chlorophyll of PS
ST: Single-turnover light pulse, with respect to PS II activity

## References

[CR78] Albertsson P-A (2001). A quantitative model of the domain structure of the photosynthetic membrane. Trends Plant Sci.

[CR1] Anderson JW, Done J (1978). Light dependent assimilation of nitrite by isolated chloroplasts. Plant Physiol.

[CR2] Arnon DI, Chain RK (1975). Regulation of ferredoxin-catalyzed photosynthetic phosphorylation. Proc Natl Acad Sci USA.

[CR3] Aronsson H, Schöttler M, Kelly AA, Sundquist C, Dörmanns P, Karim S, Jarvis P (2008). Monogalactosyldiacylglycerol deficiency in Arabidopsis affects pigment composition in the prolamellar body and impairs thylakoid membrane energization and photoprotection in leaves. Plant Physiol.

[CR4] Asada K (1999). The water-water cycle in chloroplasts: scavenging of active oxygen and dissipation of excess photons. Annu Rev Plant Physiol Plant Mol Biol.

[CR5] Asada K, Badger M (1984). Photoreduction of ^18^O_2_ and H_2_^18^O_2_ with a concomitant evolution of ^16^O_2_ in intact spinach chloroplasts: evidence for scavenging of hydrogen peroxide by peroxidase. Plant Cell Physiol.

[CR6] Asada K, Heber U, Schreiber U (1992). Pool size of electrons that can be donated to P700^+^, as determined in intact leaves: donation to P700^+^ from stromal components via the intersystem chain. Plant Cell Physiol.

[CR7] Bendall DS, Manasse RS (1995). Cyclic phosphorylation and electron transport. Biochim Biophys Acta.

[CR9] Brettel K (1997). Electron transfer and arrangement of the redox cofactors in photosystem I. Biochem Biophys Acta.

[CR10] Buchanan BB (1980). Role of light in the regulation of chloroplast enzymes. Ann Rev Plant Physiol Plant Mol Biol.

[CR11] Buchanan BB, Schürmann P, Wolosiuk RA, Jacquot J-P (2002). The ferredoxin/thioredoxin system: from discovery to molecular structures and beyond. Photosynth Res.

[CR12] Bukhov N, Egorova E, Carpentier R (2002). Electron flow to photosystem I from stromal reductants in vivo: the size of the pool of stromal reductants controls the rate of electron donation to both rapidly and slowly reducing photosystem I units. Planta.

[CR13] Carillo N, Ceccarelli EA (2003). Open questions in ferredoxin-NADP^+^ reductase catalytic mechanism. Eur J Biochem.

[CR14] Chow WS, Hope AB (2004). Electron fluxes through photosystem I in cucumber leaf discs probed by far-red light. Photosynth Res.

[CR15] Diaz-Quintana A, Leibl W, Bottin H, Setif P (1998). Electron transfer in photosystem I reaction centers follows a linear pathway in which iron-sulfur cluster *F*_B_ is the immediate electron donor to soluble ferredoxin. Biochemistry.

[CR16] Drepper F, Hippler M, Nitschke W, Haehnel W (1996). Binding dynamics and electron transfer between plastocyanin and photosystem I. Biochemistry.

[CR17] Duysens LNM, Sweers HE, Japanese Society of Plant Physiologists (1963). Mechanism of the two photochemical reactions in algae as studied by means of fluorescence. Studies on Microalgae and Photosynthetic Bacteria.

[CR18] Foyer CH, Lelandais M, Harbinson J (1992). Control of the quantum efficiencies of photosystem I and II, electron flow, and enzyme activation following dark-to-light transitions in pea leaves; relationship between NADP/NADPH ratios and NADP-malate dehydrogenase activation state. Plant Physiol.

[CR20] Golbeck JH (2006). The light-driven plastocyanin: ferredoxin oxidoreductase.

[CR21] Golding AJ, Johnson GN (2003). Down regulation of linear and activation of cyclic electron transport during drought. Planta.

[CR22] Haehnel W (1984). Photosynthetic electron transport in higher plants. Annu Rev Plant Physiol.

[CR23] Hanke G, Mulo P (2013). Plant type ferredoxins and ferredoxin-dependent metabolism. Plant Cell Environ.

[CR24] Harbinson J, Foyer CH (1991). Relationships between the efficiencies of photosystems I and II and stromal redox state in CO_2_-free air. Plant Physiol.

[CR79] Harbinson J, Hedley CL (1993). Changes in P-700 oxidation during the early stages of the induction of photosynthesis. Plant Physiol.

[CR25] Harbinson J, Woodward FI (1987). The use of light induced absorbance changes at 820 nm to monitor the oxidation state of P-700 in leaves. Plant Cell Environ.

[CR27] Hiyama T, Ke B (1971). A new photosynthetic pigment. ‘P430’: its possible role as the primary electron acceptor of Photosystem I. Proc Natl Acad Sci USA.

[CR28] Hoshina S, Itoh S, Abrol YP, Mohanty P, Govindjee A (1993). Photosystem I reaction centre in oxygenic photosynthetic organisms: current views and the future. Photosynthesis: phororeactions to plant productivity.

[CR29] Jensen PE, Bassi R, Boekema EJ, Dekker JP, Janson S, Leister D, Robinson C, Scheller HV (2007). Structure and regulation of plant photosystem I. Biochim Biophys Acta.

[CR31] Joliot P, Joliot A (1979). Comparative study of the fluorescence yield and of the C550 absorption change at room temperature. Biochim Biophys Acta.

[CR32] Joliot P, Joliot A (2002). Cyclic electron transfer in plant leaf. Proc Natl Acad Sci USA.

[CR35] Joliot P, Béal D, Joliot A (2004). Cyclic electron flow under saturating excitation of dark-adapted *Arabidopsis* leaves. Biochim Biophys Acta.

[CR37] Katoh S, Trebst A, Avron M (1977). Plastocyanin. Encyclopedia of plant physiology.

[CR38] Kautsky H, Appel W, Amann H (1960). Die Fluoreszenzkurve und die Photochemie der Pflanze. Biochem Z.

[CR39] Kirchhoff H, Schöttler MA, Maurer J, Weis E (2004). Plastocyanin redox kinetics in spinach chloroplasts: evidence for disequilibrium in the high potential chain. Biochim Biophys Acta.

[CR40] Klughammer C (1992) Entwicklung und Anwendung neuer absorptionsspektroskopischer Methoden zur Charakterisierung des photosynthetischen Elektronentransports in isolierten Chloroplasten und intakten Blättern. Ph.D. Thesis, University of Würzburg, Germany

[CR41] Klughammer C, Schreiber U (1991). Analysis of light-induced absorbance changes in the near-infrared spectral region. I. Characterization of various components in isolated chloroplasts. Z Naturforsch C.

[CR43] Klughammer C, Schreiber U (1994). An improved method, using saturating light pulses, for the determination of photosystem I quantum yield via P700+-absorbance changes at 830 nm. Planta.

[CR44] Klughammer C, Schreiber U, Garab G (1998). Measuring P700 absorbance changes in the near infrared spectral region with a dual wavelength pulse modulation system. Photosynthesis: mechanisms and effects.

[CR45] Klughammer C, Kolbowski J, Schreiber U (1990). LED array spectrophotometer for measurement of time resolved difference spectra. Photosynth Res.

[CR46] Knaff DB, Ort DR, Yocum CF (1996). Ferredoxin and ferredoxin-dependent enzymes. Advances in Photosynthesis.

[CR47] Laisk A, Oja V, Heber U (1992). Steady-state and induction kinetics of photosynthetic electron transport related to donor side oxidation and acceptor side reduction of Photosystem I in sunflower leaves. Photosynthetica.

[CR48] Laisk A, Talts E, Oja V, Eichelmann H, Peterson RB (2010). Fast cyclic electron transport around photosystem I in leaves under far-red light: a proton-uncoupled pathway?. Photosynth Res.

[CR49] Malkin R, Bearden AJ (1971). Primary reaction of photosynthesis: photoreduction of a bound chloroplast ferredoxin at low temperatures as detected by EPR spectroscopy. Proc Natl Acad Sci USA.

[CR50] Melis A, Duysens LNM (1979). Biphasic energy conversion kinetics and absorbance difference spectra of Photosystem II of chloroplasts. Evidence for two different PS II reaction centers. Photochem Photobiol.

[CR51] Melis A, Schreiber U (1979). The kinetic relationship between the C-550 absorbance change, the reduction of *Q*_A_ (A_320_) and the variable fluorescence yield change in chloroplasts at room temperature. Biochim Biophys Acta.

[CR52] Miyake C (2010). Alternative electron flows (water–water cycle and cyclic electron flow around PSI) in photosynthesis: molecular mechanisms and physiological functions. Plant Cell Physiol.

[CR53] Miyake C, Schreiber U, Asada K (1995). Ferredoxin-dependent and antimycin A-sensitive reduction of cytochrome b-559 by far-red light in maize thylakoids; participation of a menadiol-reducible cytochrome b-559 in cyclic electron flow. Plant Cell Physiol.

[CR80] Munekage Y, Hojo M, Meurer J, Endo T, Tasaka M, Shikanai T (2002). PGR5 is involved in cyclic electron flow around photosystem I and is essential for photoprotection in Arabidopsis. Cell.

[CR54] Neubauer C, Schreiber U (1987). The polyphasic fluorescence rise of chlorophyll fluorescence upon onset of strong continuous illumination: I. Saturation characteristics and partial control by the Photosystem II acceptor side. Z Naturforsch C.

[CR91] Oh-Oka H, Itoh S, Saeki K, Takahashi Y, Matsubara H (1991). F_A_/F_B_ protein from the spinach photosystem I complex: Isolation in a native state and some properties of the iron-sulfur clusters. Plant Cell Physiol.

[CR55] Oja V, Eichelmann H, Peterson RB, Laisk A (2003). Deciphering the 820 nm signal: redox state of donor side and quantum yield of photosystem I in leaves. Photosynth Res.

[CR56] Oja V, Bichele I, Hüve K, Rasulov B, Laisk A (2004). Reductive titration of photosystem I and differential extinction coefficient of P700^+^ at 810–950 nm in leaves. Biochim Biophys Acta.

[CR57] Papageorgiou GC, Govindjee (2004). Chlorophyll a fluorescence: a signature of photosynthesis.

[CR58] Sacksteder CA, Kramer DA (2000). Dark interval relaxation kinetics (DIRK) of absorbance changes as a quantitative probe of steady-state electron transfer. Photosynth Res.

[CR59] Schansker G, Srivastava A, Govindjee, Strasser RJ (2003). Characterization of the 820-nm transmission signal paralleling the chlorophyll a fluorescence rise (OJIP) in pea leaves. Funct Plant Biol.

[CR60] Schöttler MA, Toth SZ (2014). Photosynthetic complex stoichiometry dynamics in higher plants: environmental acclimation and photosynthetic flux control. Front Plant Sci.

[CR61] Schöttler MA, Flügel C, Thiele W, Bock R (2007). Knock-out of the plastid-encoded PetL subunit results in reduced stability and accelerated leaf age-dependent loss of the cytochrome b_6_f complex. J Biol Chem.

[CR62] Schöttler MA, Flügel C, Thiele W, Stegemann S, Bock R (2007). The plastome-encoded PsaJ subunit is required for efficient photosystem I excitation, but not for plastocyanin oxidation in tobacco. Biochem J.

[CR63] Schreiber U (1986). Detection of rapid induction kinetics with a new type of high-frequency modulated chlorophyll fluorometer. Photosynth Res.

[CR64] Schreiber U, Neubauer C (1987). The polyphasic fluorescence rise of chlorophyll fluorescence upon onset of strong continuous illumination: II. Partial control by the Photosystem II donor side and possible ways of interpretation. Z Naturforsch C.

[CR66] Schreiber U, Klughammer C, Neubauer C (1988). Measuring P700 absorbance changes around 830 nm with a new type of pulse modulation system. Z Naturforsch C.

[CR67] Schreiber U, Neubauer C, Klughammer C (1989). Devices and methods for room-temperature fluorescence analysis. Phil Trans R Soc Lond B.

[CR68] Schreiber U, Hormann H, Asada K, Neubauer C, Mathis P (1995). O_2_-dependent electron flow in spinach chloroplasts: properties and possible regulation of the Mehler-ascorbate peroxidase cycle. Photosynthesis: From Light to Biosphere.

[CR92] Schürmann P, Buchanan BB (2008). The ferredoxin/thioredoxin system of oxygenic photosynthesis. Antioxid Redox Signal.

[CR69] Sétif P (2001). Ferredoxin and flavodoxin reduction by photosystem I. Biochim Biophys Acta.

[CR70] Sétif P, Bottin H (1995). Laser flash absorption spectroscopy strudy of ferredoxin reduction by photosystem I: spectral and kinetic evidence for the existence of several photowystem I-ferredoxin complexes. Biochemistry.

[CR71] Shin M, Tagawa K, Arnon DI (1963). Crystallization of ferredoxin-TPN reductase and its role in photosynthetic apparatus of chloroplasts. Biochem Z.

[CR72] Siebke K, Laisk A, Neimanis S, Heber U (1991). Regulation of chloroplast metabolism in leaves: evidence that NADP-dependent glyceraldehydephosphate dehydrogenase, but not ferredoxin-NADP reductase, controls electron flow to phosphoglycerate in the dark-light transition. Planta.

[CR73] Stirbet A, Govindjee (2012). Chlorophyll a fluorescence induction: a personal perspective of the thermal phase, the J-I-P rise. Photosynth Res.

[CR74] Strasser RJ, Srivastava A, Govindjee (1995). Polyphasic chlorophyll *a* fluorescence transient in plants and cyanobacteria. Photochem Photobiol.

[CR75] Talts E, Oja V, Rämma H, Rasulov B, Anijalg A, Laisk A (2007). Dark inactivation of ferredoxin-NADP reductase and cyclic electron flow under far-red light in sunflower leaves. Photosynth Res.

[CR76] Vassiliev IR, Jung YS, Yang F, Golbeck JH (1998). PsaC subunit of photosystem I is oriented with iron-sulfur cluster *F*_B_ as immediate electron donor to ferredoxin and flavodoxin. Biophy J.

[CR77] Weis E, Ball JT, Berry JA, Biggins J (1987). Photosynthetic control of electron transport in leaves of Phaseolus vulgaris: evidence for regulation of photosystem II by the proton gradient. Progress in photosynthesis research.

