# Supplementary material for: Deconvolution of ferredoxin, plastocyanin, and P700 transmittance changes in intact leaves with a new type of kinetic LED array spectrophotometer
Source: Photosynth Res. 2016 Feb 2;128:195–214. doi: 10.1007/s11120-016-0219-0 (PMC4826414; doi:10.1007/s11120-016-0219-0)
Supplement: Supplementary file 1 — Supplementary material 1 (PDF 378 kb) [file 11120_2016_219_MOESM1_ESM.pdf]

Supplementary Figures (Klughammer and Schreiber PRES-D-15-00130)

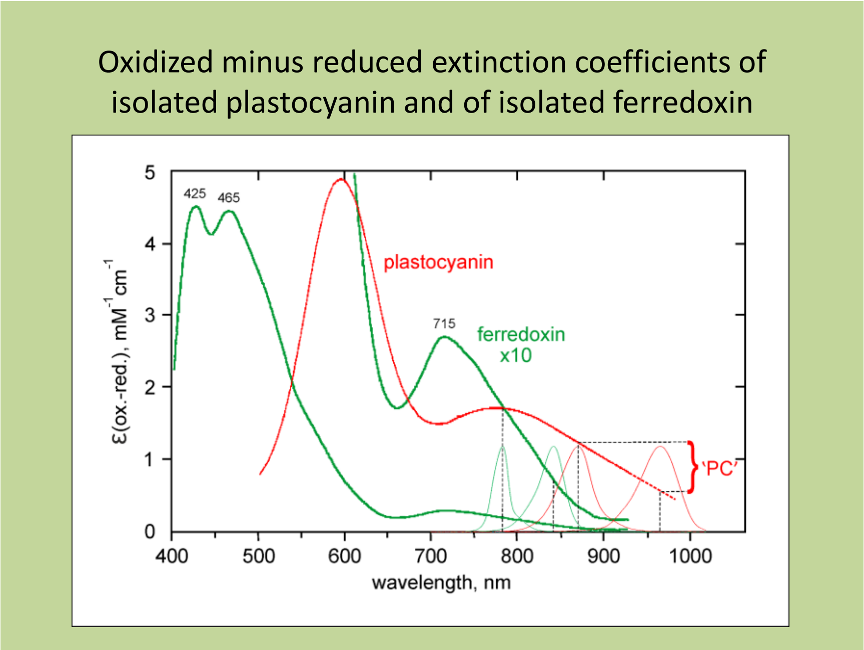

Supplementary Figure 1 Oxidized minus reduced extinction coefficients of isolated PC and Fd. Reproduced from Klughammer (1992). While absorbance by both PC^+ and Fd declines at wavelengths > 785 nm, the decline of Fd absorbance is distinctly steeper. Emission spectra of the wavelength pairs 785 – 840 nm ('Fd', green) and 870 – 970 nm ('PC', orange) are inserted.

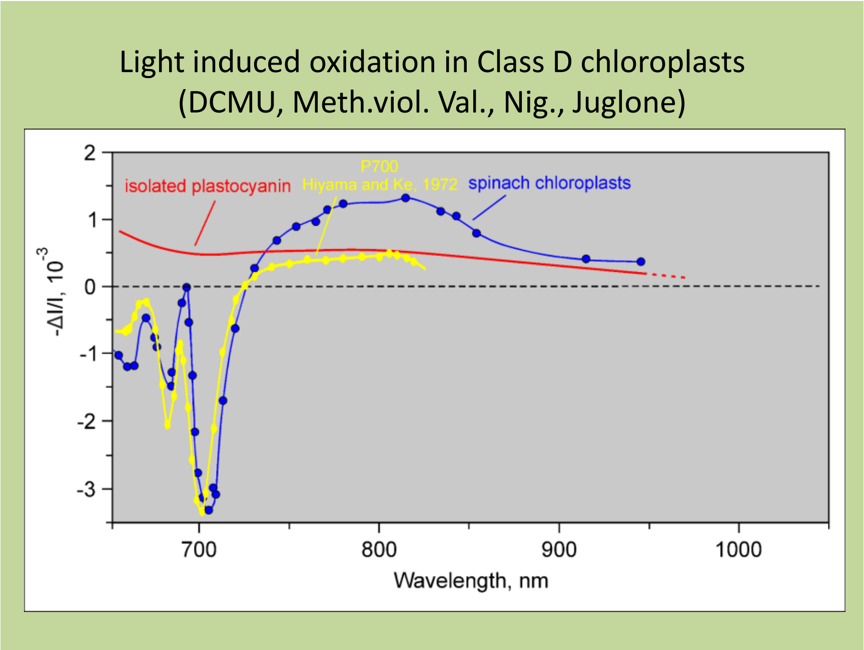

Supplementary Figure 2 Transmittance changes induced by light induced oxidation in broken spinach chloroplasts compared with oxidized minus reduced difference spectrum of isolated PC (see also Supplementary Figure 1) and the P700 difference spectrum of PS I particles published by Hiyama and Ke (1972). Reproduced from Klughammer (1992). $P700^+$ absorbance displays a broad peak around 810 nm and then declines distinctly steeper than PC^+ absorbance.

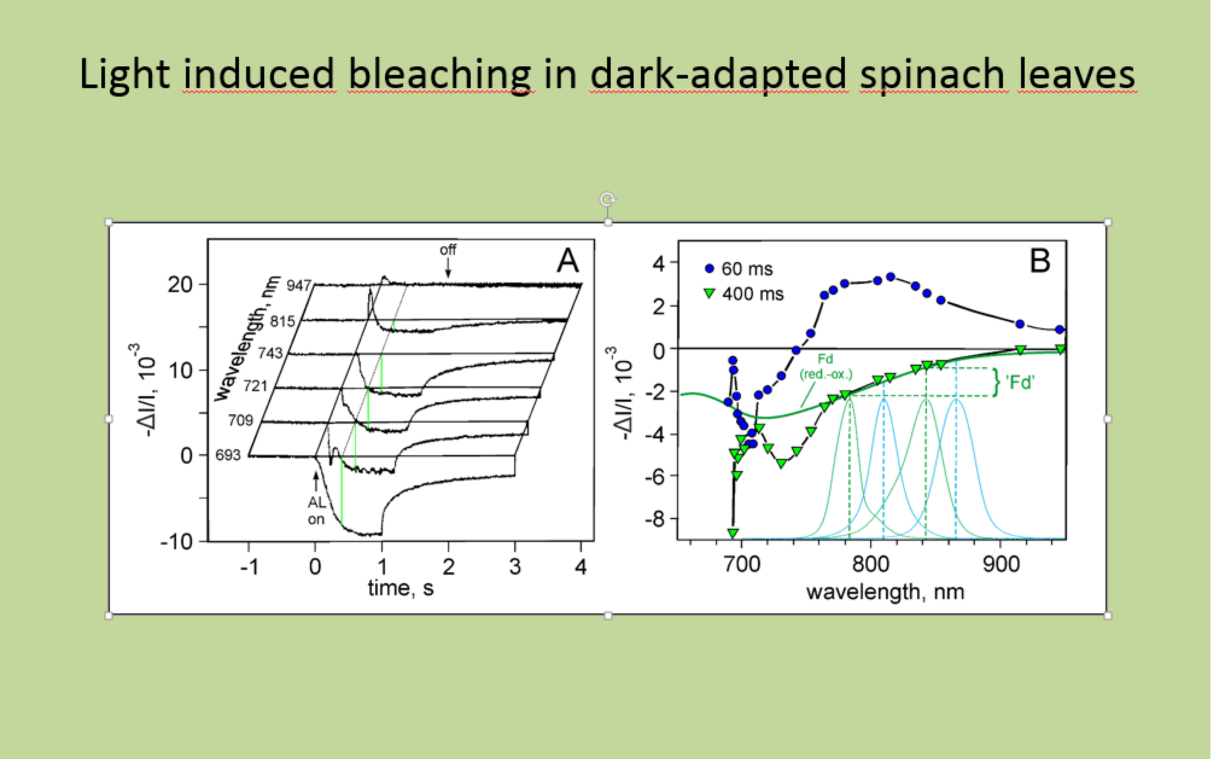

Supplementary Figure 3 Blue light induced single wavelength transmittance changes of dark-acclimated spinach leaves in the far-red and NIR spectral region. Reproduced from Klughammer (1992). While the initial change at 60 ms is mainly due to P700 and PC oxidation, the change at 400 ms is dominated by Fd reduction. Bleaching by reduced Fd declines between 785 and 840 nm, where the absorbance increase due to P700+PC oxidation is almost constant. Therefore, the 785 – 840 nm difference signal (‘Fd’) is relatively specific for Fd redox changes.

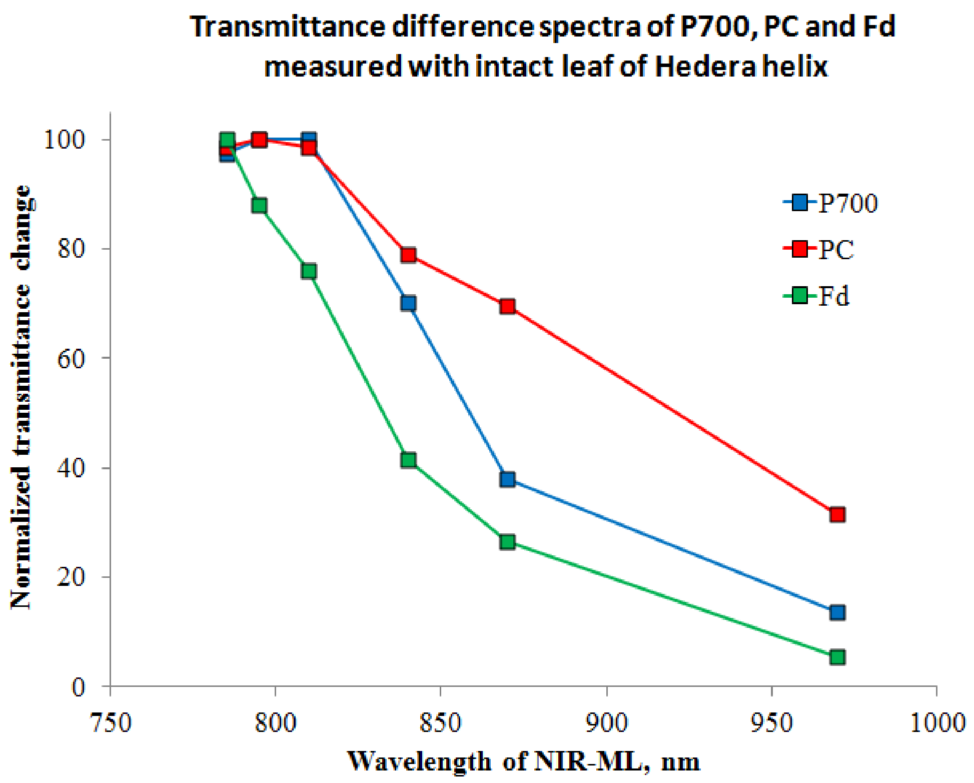

Supplementary Figure 4 Single wavelength transmittance changes in intact leaf of *Hedera helix* induced under conditions favoring *selective* redox changes of either P700 or PC or Fd. Maximal reduced minus oxidized (P700 and PC) and oxidized minus reduced (Fd) transmittance changes were normalized to 100 units. Conditions were essentially as described under 'Materials and methods' (section on 'Selective changes and Differential Model Plots') and as applied for the dual-wavelength measurements of Figs. 6-8:

For selective P700: Application of 5 µs ST in presence of FR background light; sampling window at 0.5 s following ST; average of 25 measurements at 785, 795, 810 and 870 nm as well as 25 measurements at 840 and 970 nm.

For selective PC: Application of 50 µs ST upon termination of FR illumination; sampling window at 5 s following ST and FR-off; average of 10 measurements at 785, 795, 810 and 870 nm as well as 10 measurements at 840 and 970 nm.

For selective Fd: 0.6 s illumination of dark-acclimated leaf; sampling window at 1 s following AL-off; average of 16 measurements at 785, 795, 810 and 870 nm as well as 16 measurements at 840 and 970 nm.

Measurements were carried out using the new Dual/KLAS-NIR spectrophotometer in the single wavelength mode of operation. For this purpose either the LED currents of all longer wavelengths or of all shorter wavelengths of the four wavelength-pairs were manually set to zero. All currents of the active LEDs were manually adjusted to 3V and then compensated to zero.

These *in vivo* difference spectra may serve to illustrate the choice of wavelength pairs for measuring 'Fd-enriched' (785 – 840 nm), 'P700-enriched' (810-870 nm) and 'PC-enriched' (870 – 970 nm) transmittance changes. For obtaining genuine Fd, P700 and PC changes deconvolution is required.
